# The Rhoptry Proteins ROP18 and ROP5 Mediate *Toxoplasma gondii* Evasion of the Murine, But Not the Human, Interferon-Gamma Response

**DOI:** 10.1371/journal.ppat.1002784

**Published:** 2012-06-28

**Authors:** Wendy Niedelman, Daniel A. Gold, Emily E. Rosowski, Joris K. Sprokholt, Daniel Lim, Ailan Farid Arenas, Mariane B. Melo, Eric Spooner, Michael B. Yaffe, Jeroen P. J. Saeij

**Affiliations:** 1 Department of Biology, Massachusetts Institute of Technology, Cambridge, Massachusetts, United States of America; 2 Department of Cell Biology and Immunology, Wageningen University and Research Centre, Wageningen, The Netherlands; 3 Koch Institute for Integrative Cancer Research, Massachusetts Institute of Technology, Cambridge, Massachusetts, United States of America; 4 Group of Molecular Parasitology, Universidad del Quindio, Quindio, Colombia; 5 Whitehead Institute for Biomedical Research, Cambridge, Massachusetts, United States of America; University of Geneva, Switzerland

## Abstract

The obligate intracellular parasite *Toxoplasma gondii* secretes effector proteins into the host cell that manipulate the immune response allowing it to establish a chronic infection. Crosses between the types I, II and III strains, which are prevalent in North America and Europe, have identified several secreted effectors that determine strain differences in mouse virulence. The polymorphic rhoptry protein kinase ROP18 was recently shown to determine the difference in virulence between type I and III strains by phosphorylating and inactivating the interferon-γ (IFNγ)-induced immunity-related GTPases (IRGs) that promote killing by disrupting the parasitophorous vacuole membrane (PVM) in murine cells. The polymorphic pseudokinase ROP5 determines strain differences in virulence through an unknown mechanism. Here we report that ROP18 can only inhibit accumulation of the IRGs on the PVM of strains that also express virulent *ROP5* alleles. In contrast, specific *ROP5* alleles can reduce IRG coating even in the absence of *ROP18* expression and can directly interact with one or more IRGs. We further show that the allelic combination of *ROP18* and *ROP5* also determines IRG evasion and virulence of strains belonging to other lineages besides types I, II and III. However, neither ROP18 nor ROP5 markedly affect survival in IFNγ-activated human cells, which lack the multitude of IRGs present in murine cells. These findings suggest that ROP18 and ROP5 have specifically evolved to block the IRGs and are unlikely to have effects in species that do not have the IRG system, such as humans.

## Author Summary

*Toxoplasma gondii* can infect any warm-blooded animal and is transmitted orally by consumption of tissue cysts. To facilitate transmission, the parasite must balance induction and evasion of host immune responses to allow parasite growth and persistence, while avoiding excessive parasite burden, which can kill the host before infectious cysts are formed. Different strains of *Toxoplasma* have likely evolved specific effector molecules to modulate the immune responses of different hosts. In many mammals, including mice but not humans, the cytokine interferon gamma (IFNγ) induces the immunity-related GTPases (IRGs), which are essential to the murine immune response to *Toxoplasma*. They function by binding to and disrupting the parasite-containing vacuole. However, some *Toxoplasma* strains prevent the IRGs from disrupting the parasitophorous vacuole. It was previously shown that the secreted *Toxoplasma* kinase ROP18 promotes virulence in mice by phosphorylating the IRGs, leading to their inactivation. We report that ROP18 requires another virulence factor, the secreted pseudokinase ROP5, to prevent IRG accumulation, and these two proteins determine the majority of strain differences in IRG evasion, even for divergent strains for which virulence determinants have not been studied. Additionally, we show that ROP18 and ROP5 do not affect *Toxoplasma* survival in IFNγ-stimulated human cells. Thus, ROP18 and ROP5 are strain- and host-specific determinants of *Toxoplasma* immune evasion.

## Introduction

*Toxoplasma gondii* is a widespread intracellular parasite capable of infecting most warm-blooded animals and is an important opportunistic pathogen for immunocompromised individuals and unborn fetuses. *Toxoplasma* resides within a non-fusogenic parasitophorous vacuole and has three apical secretory organelles, the micronemes, rhoptries and dense granules, which secrete proteins into the host cell during invasion that mediate important host-pathogen interactions [1]. In general, an asymptomatic but chronic infection is established in immunocompetent humans. However, in rare cases *Toxoplasma* can cause severe disease even in immunocompetent people. Diverse disease outcomes may be due to genetic differences between infecting strains [2].

*Toxoplasma* has a partially clonal population structure of 12–15 [3], [4] haplogroups with the majority of North American and European isolates belonging to the canonical types I, II and III strains [5], [6], although haplogroup 12 has been recently shown to be prevalent in wild animals in North America [6]. In mice, these strains differ in virulence, with type I strains having an LD_100_ of just one parasite, compared to the LD_50_ of ∼10^3^ or ∼10^5^ parasites for types II and III strains, respectively [7], [8]. Type I strains may also be more virulent in humans, as they are more frequently isolated from cases of congenital or severe ocular toxoplasmosis than from animals [5], [9]. Interestingly, in South America, more genetically diverse strains are isolated, while the canonical strains are rarely found [10]. Some of these strains are associated with high mortality rates in mice [11]. Additionally, there are high rates of ocular toxoplasmosis in humans in South America [12], [13], and some strains isolated from French Guiana have been reported to cause severe disseminated toxoplasmosis even in healthy individuals [14]. The determinants of canonical strain-specific differences in murine virulence are well studied, but the same determinants for non-canonical strains or for human infection remain unknown.

Mice and humans use divergent immune mechanisms to resist *Toxoplasma*. Interferon-γ (IFNγ) is essential to murine *Toxoplasma* resistance, and IFNγ-deficient mice die after infection even with avirulent strains [15]. Some of the important downstream effectors of this immune activation are the IFNγ-inducible immunity-related GTPases (IRGs), which belong to the dynamin family of GTPases and can cooperatively oligomerize to vesiculate membranes. Mice deficient in individual members of the IRG family die of toxoplasmosis, but at different stages of infection, and expression of the IRGs is required even in non-hematopoietic cells, suggesting IRGs have non-redundant, crucial roles in the innate immune response against *Toxoplasma* [16]–[18]. Different IRGs are sequentially and cooperatively loaded onto the parasitophorous vacuole membrane (PVM) with Irgb6 and Irgb10 initiating and stabilizing the loading of the other members [19]. The IRGs are able to disrupt the PVM and kill the parasite [20], [21].

While mice have 23 IRG genes, humans have only two IRG genes: *IRGC* which is expressed only in the testis and *IRGM* which is expressed independently of IFNγ induction and has a truncation in the nucleotide-binding G-domain [22]. Despite these differences, IRGM plays a role in autophagy-mediated destruction of *Mycobacterium tuberculosis* and *Salmonella typhimurium* in human cells, and some variants are associated with increased risk for Crohn's disease [23], [24]. Thus, IRGM may have an immune role, but its lack of GTPase activity suggests a distinct mechanism of action in humans. Humans do have other known IFNγ-mediated mechanisms of resistance to *Toxoplasma*. For instance, IFNγ-induced indoleamine 2,3-dioxygenase (IDO1) degrades cellular tryptophan for which *Toxoplasma* is auxotrophic, thereby inhibiting *Toxoplasma* growth [25], [26]. The NALP1 inflammasome also mediates the innate immune response to *Toxoplasma*, and *NALP1* was recently identified as a susceptibility locus for human congenital toxoplasmosis [27].

*Toxoplasma* strain differences in evasion of murine immune responses exist. For instance, type I strains are able to prevent the accumulation of IRGs on the PVM, while types II and III strains are susceptible to killing by the IRGs even when co-infecting the same cell as a type I parasite [28]. Because strain-specific evasion of the IRGs is correlated with increased virulence in the mouse, it is likely that the genetic determinants of IRG evasion will also be associated with virulence. Quantitative Trait Locus (QTL) mapping analyses of the virulence of F1 progeny derived from type I×II, I×III and II×III crosses have identified the genetic loci associated with virulence, and subsequent experiments have identified the causative genes within these loci.

ROP18, a highly polymorphic rhoptry protein kinase, was identified as a virulence locus in the II×III QTL study and the only virulence locus in the I×III cross [7], [29]. ROP18 is highly expressed in types I and II strains but an insertion in the promoter prevents expression in type III strains. Addition of a type I or II copy of *ROP18* into an avirulent type III strain makes that strain become virulent [7], [11]. Recently, it was shown that type I ROP18 can phosphorylate a conserved threonine in the G-domain of Irga6 and Irgb6, disrupting their accumulation on the PVM [30], [31]. However, type II strains have the highest percentage of vacuoles coated with IRGs [19], [28] despite the fact that a type II copy of ROP18 is also able to make a type III strain virulent, suggesting that other polymorphic proteins are involved in IRG evasion [7]. ROP18 was also shown to promote the degradation of the endoplasmic reticulum-associated transcription factor ATF6-β, compromising CD8 T cell-mediated adaptive immune responses [32]. Importantly, ROP18-mediated ATF6-β degradation occurs in human as well as murine cells.

The *ROP5* locus, which consists of a family of 4–10 tandem duplicates of highly polymorphic genes encoding for rhoptry pseudokinases that localize to the PVM, is another important virulence determinant in mice [33], [34]. Deletion of *ROP5* in a type I strain significantly attenuates virulence. Furthermore, *ROP5* was the only significant virulence locus identified in the recent I×II QTL analysis and was the main virulence locus in the II×III QTL study [7], [34]. Both types I and III strains have a virulent *ROP5* locus, but the mechanism by which ROP5 affects virulence and which of the three major ROP5 isoforms, A, B or C, [33] are necessary to complement the virulence of type II are not known.

A third virulence locus, identified in the II×III QTL study, contains the rhoptry protein kinase ROP16, which in types I and III strains leads to sustained phosphorylation and activation of STAT3/6 [35]. It was recently shown that ROP16 and the dense granule protein GRA15, suggested to be the fourth virulence locus in the II×III QTL study [36], affect the accumulation of p65 guanylate binding proteins (GBPs) on the PVM in infected murine cells [37]. Because GBPs are also dynamin family members and were found on the same vacuoles as the IRGs, ROP16 and GRA15 might also affect the accumulation of the IRGs on the PVM. Furthermore, since the GBPs are present in humans, ROP16 and GRA15 could possibly affect survival in IFNγ-stimulated human cells.

Because the murine and human immune responses to *Toxoplasma* are so different, it cannot be assumed that ROP18, ROP5, ROP16 and GRA15, which determine *Toxoplasma* virulence in mice, similarly affect survival in human cells. Furthermore, it is currently unknown for most of these proteins what effects they have outside the clonal lineages from which they were identified. Many of the exotic strains are highly virulent in mice, but because they are so divergent from the canonical strains and the exotic strains have not been used in QTL or gene manipulation studies, it is not known what factors drive virulence in these strains. For example, IRG evasion has not been measured for the exotic strains, and it may be that this is strictly a type I phenotype.

In this study, we find that ROP18 can only inhibit accumulation of the IRGs on the PVM of strains that also express virulent *ROP5* alleles. Expression of *ROP18* in strains that do not express virulent *ROP5* alleles does not affect IRG accumulation or *in vivo* virulence. In contrast, specific *ROP5* alleles can reduce IRG coating even in the absence of *ROP18* expression and directly interact with Irga6 to inhibit its oligomerization. Non-canonical strains exhibit differences in evasion of IRG-mediated killing as well, and the allelic combination of *ROP18* and *ROP5* also correlates with strain differences in IRG evasion and virulence for these strains. However, neither ROP18 nor ROP5 markedly affect parasite survival in IFNγ-activated human cells.

## Results

### Both ROP18_II_ and ROP18_I_ reduce IRG-mediated killing of type III parasites

**Figure 1:**
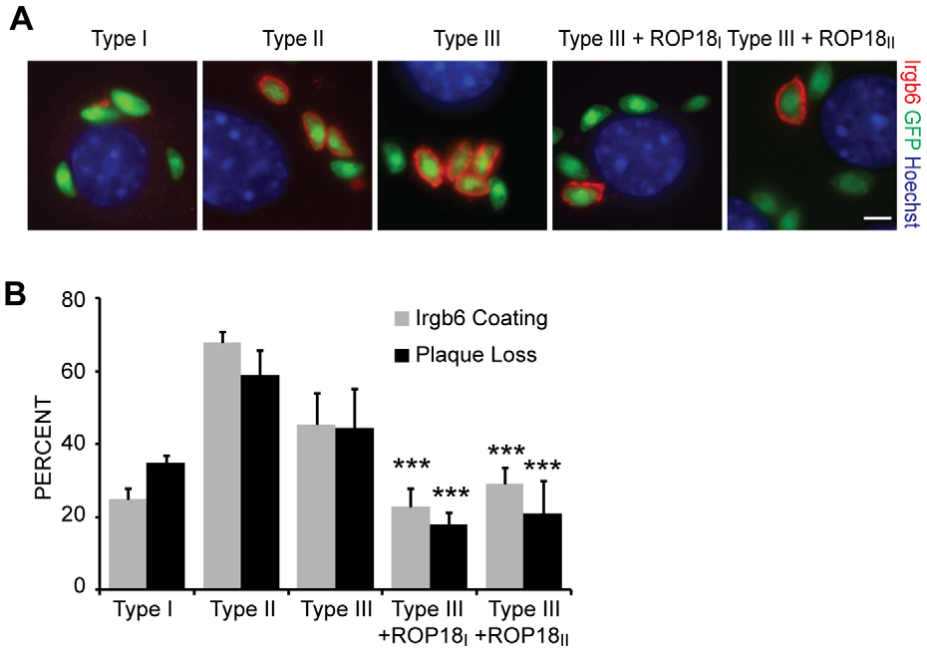
A type III strain expressing type I or type II ROP18 inhibits Irgb6 accumulation and killing. WT MEFs were stimulated for 24 hours with IFNγ and infected with type I (GT1), type II (Pru), type III (CEP), CEP + ROP18_I_ or CEP + ROP18_II_ expressing GFP. (A) Cells were fixed after 1 hour and stained by immunofluorescence for Irgb6 (Red) and with Hoechst (blue). Scale bar represents 5 µm. (B) Quantification of Irgb6 localization on the parasite containing vacuole and percentage plaque loss after 4–7 days on stimulated MEFs compared to unstimulated MEFs. Mean + SEM, n = 5 experiments, ***p<0.001, Student's t test.

Type II strains have the highest percentage of IRG-coated vacuoles compared to types I and III strains [19], [28] even though they possess a *ROP18* allele capable of conferring virulence to a type III strain [7]. To determine if, like ROP18_I_ [30], [31], the increased virulence due to ROP18_II_ is correlated with reduced IRG coating in a type III background, we measured the percentage of vacuoles coated with Irgb6 by immunofluorescence in IFNγ-stimulated mouse embryonic fibroblasts (MEFs) infected with type I, II, III, III + ROP18_I_, or III + ROP18_II_ (Figure 1A). Indeed, transgenic expression in the type III strain CEP of either *ROP18_I_* or *ROP18_II_* decreased the average number of vacuoles coated with Irgb6 from 45% to 23% (P = 0.001) for ROP18_I_ or 29% (P = 0.003) for ROP18_II_ (Figure 1B). Although it is generally assumed that once the PVM is coated, it will eventually lead to killing of the parasite inside, it has also been shown that *Toxoplasma* can escape a coated vacuole and invade a new cell [37], [38]. Therefore, to measure killing of *Toxoplasma*, 100 parasites were seeded on a monolayer of MEFs, either previously stimulated for 24 hours with IFNγ or left untreated, and the number of plaques that form after 4–7 days of growth was determined. Type III had an average of 45% plaque loss when comparing plaques formed on IFNγ-stimulated MEFs to unstimulated MEFs. This percentage plaque loss was similar to the percentage of vacuoles coated with Irgb6, suggesting that coated vacuoles are eventually destroyed. Furthermore, plaque loss is drastically reduced in Atg7 deficient MEFs (Figure S1) in which the IRGs are misregulated as previously reported for Atg5 deficient MEFs [19], [39], suggesting the killing observed is indeed due to the IRGs. Similar to the decrease in Irgb6 coating, the plaque loss of type III + *ROP18_I_* or *ROP18_II_* was significantly decreased to 18% (P = 0.0002) and 21% (P = 0.0004), respectively (Figure 1B). The 23% PVM coating and 18% killing of type III + *ROP18_I_* is similar to the 25% coating and 35% plaque loss of the type I strain GT1. Thus, ROP18 expression can likely explain most of the difference in IRG coating and killing between type I and type III strains. Despite the ability of ROP18_II_ to reduce IRG coating of type III strain vacuoles and subsequent killing of the parasite, type II strains are still very susceptible to the IRGs, with 70% Irgb6 coating and 73% plaque loss for Pru (type II) (Figure 1B). Thus, there must be at least one other gene involved in IRG evasion that is shared between types I and III but different in type II.

### ROP5 reduces IRG-mediated killing

**Figure 2:**
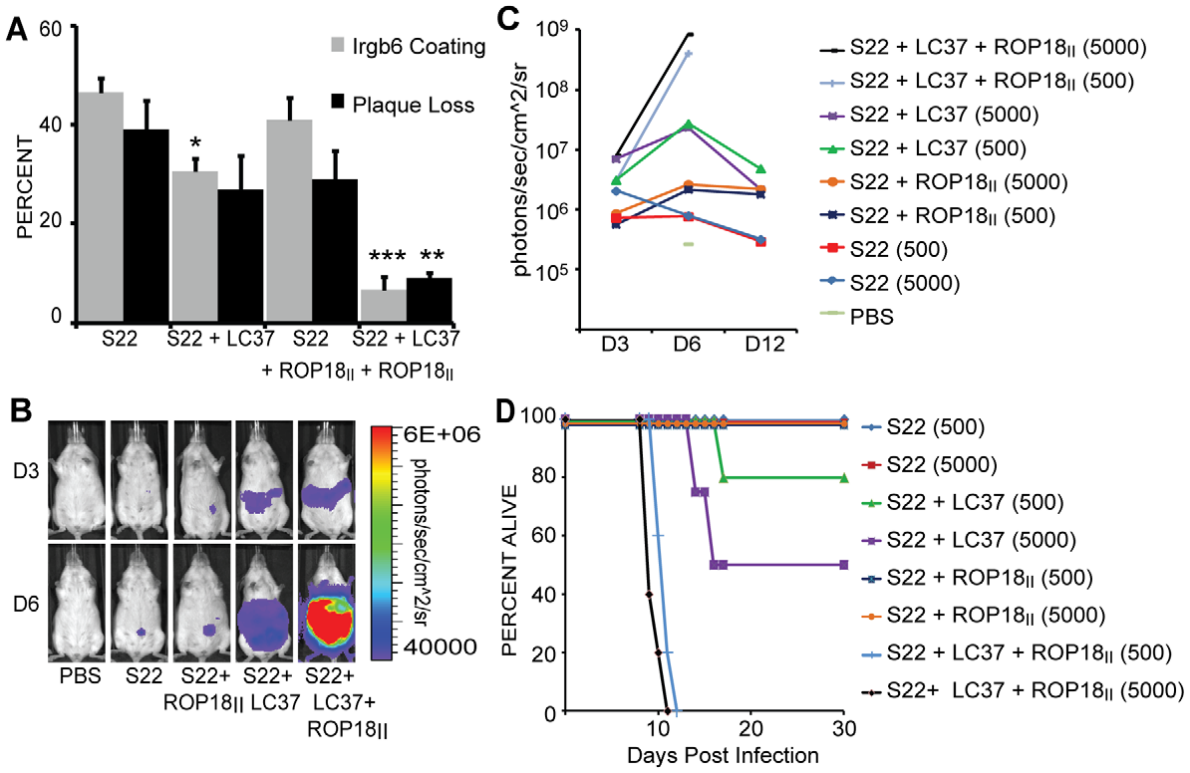
Virulent ROP5 promotes IRG evasion, independently of ROP18. (A) Quantification of Irgb6 localization on the parasitophorous vacuole (PV) and percentage plaque loss on IFNγ-stimulated MEFs compared to unstimulated MEFs infected with S22, S22 + ROP18_II_, S22 + LC37 and S22+ LC37 + ROP18_II_. Mean + SEM, n>4 experiments, *p<0.05, **p<0.01, ***p<0.001, One-way ANOVA with Bonferroni correction for all pairwise comparisons. (B) *In vivo* imaging of mice infected with firefly luciferase-expressing parasites of the indicated strains at days 3 and 6 post infection with 5000 parasites. One representative of 5 infected mice per strain is shown. (C) Quantification of *in vivo* imaging shown as average photons/sec/cm^2^/sr for infected mice at days 3, 6 and 12. (D) Mouse survival of infection with indicated doses of each strain.

It was recently demonstrated that the *ROP5* cluster of pseudokinases accounts for most of the variation in virulence between types I and II strains and between types II and III strains, with types I and III strains possessing a virulent *ROP5* locus [33], [34]. Therefore, the *ROP5* locus is an excellent candidate for explaining strain differences in IRG evasion. We tested a potential role of ROP5 in mediating ROP18-independent strain differences in IRG evasion by using the S22 strain, an avirulent F1 progeny from a II×III cross [40] which possesses the avirulent *ROP18_III_* and *ROP5_II_* alleles. We compared the percentage plaque loss and percentage of Irgb6 coated vacuoles between S22 and an S22 transgenic strain carrying the cosmid LC37, which contains the *ROP5* locus from the RH (type I) genome and was previously shown to have significantly increased virulence [33]. Expression of ROP5_I_ significantly reduced the Irgb6 coating from 48% to 28% (P<0.001), and the plaque loss from 38% to 27% (n.s.) (Figure 2A). Thus, ROP5_I_ can function independently of ROP18_I/II_ to prevent IRG accumulation on the PVM and subsequent killing of the parasite.

### ROP18_II_ requires ROP5 to reduce IRG coating

While ROP5 can function independently of ROP18 in reducing IRG accumulation on the PVM of S22 + LC37 vacuoles, type II strains, which have a virulent allele of *ROP18* and an avirulent *ROP5* locus, have a high percentage of IRG-coated vacuoles. This suggests that either ROP18 cannot function independently of ROP5, or that ROP18 is inhibited in the type II background. We expressed *ROP18_II_* in S22 and in S22 + LC37 to determine if ROP18_II_ can function in the absence of virulent ROP5 alleles. ROP18_II_ only slightly reduced Irgb6 coating in S22 from 47% to 41% (n.s.) and plaque loss from 39% to 24% (n.s.). However, ROP18_II_ significantly reduced Irgb6 coating from 31% to 7% (P<0.001) and plaque loss from 27% to 9% (P<0.01) when expressed in S22 + LC37 (Figure 2A). Together, this suggests that ROP18 needs the virulent *ROP5* locus for its function. That the Irgb6 coating and plaque loss in S22 + LC37 + ROP18_II_ are similar to those in RH (type I) signifies that these two genes are sufficient to complement IRG evasion and plaque loss in the S22 background.

To determine if the interactive effect of ROP18 and ROP5 on parasite survival also occurs *in vivo*, we infected outbred CD-1 mice by intraperitoneal injection with S22, S22 + ROP18_II_, S22 + LC37 or S22 + LC37 + ROP18_II_ tachyzoites expressing firefly luciferase and followed parasite growth and dissemination using *in vivo* imaging. On the third day after infection, the parasite burden in S22 + LC37 and S22 + LC37 + ROP18_II_-infected mice was 10-fold higher than in S22 or S22 + ROP18_II_-infected mice. By day six, both strains containing the LC37 cosmid had disseminated throughout the peritoneal cavity, but S22 + LC37 + ROP18_II_-infected mice had 35-fold higher luciferase activity than S22 + LC37-infected mice (P = 0.03), which in turn had 10-fold higher activity than S22 + ROP18_II-_infected mice (P = 0.1) and 30-fold higher activity than S22-infected mice (P = 0.06). While S22 + ROP18_II_ had a greater parasite burden than S22, this was not significant (P = 0.27). Indeed, S22 + LC37 + ROP18_II_ killed 100% of the mice in the acute stage of infection at both a low and high dose (Figure 2B and C). Likewise, in keeping with the increased IRG evasion of S22 + LC37 but not S22 + ROP18_II_, S22 + LC37 showed increased virulence compared to S22, but S22 + ROP18_II_-infected mice survived the infection and did not show significant differences compared to S22 infected mice (Figure 2D). Thus, overall these results suggest that ROP18 only affects virulence in the context of a virulent *ROP5* locus.

### IRG evasion differences in non-canonical strains

**Figure 3:**
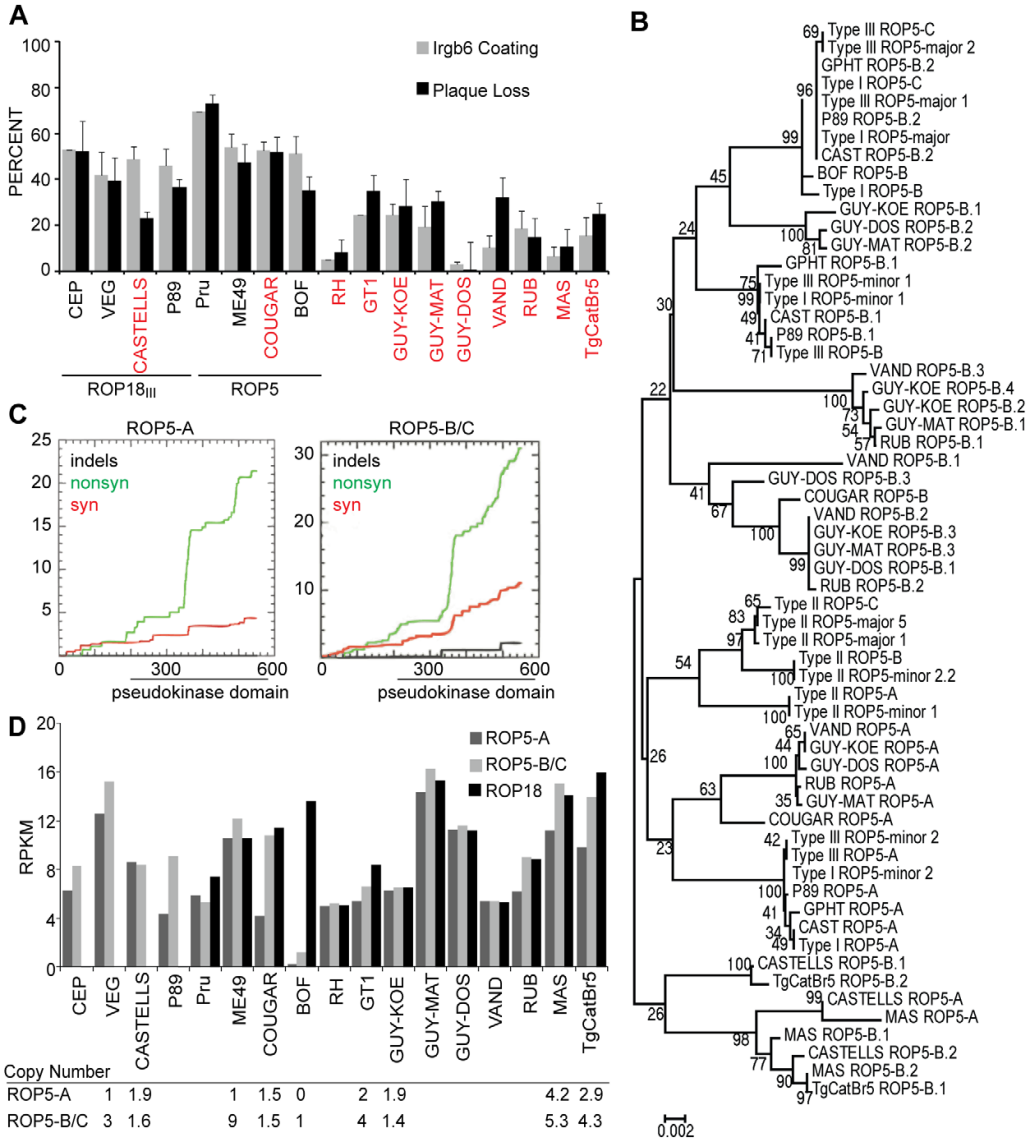
ROP5-A, ROP5-B/C and ROP18 account for strain differences in IRG evasion in non-canonical strains. (A)Quantification of percentage of PVs with Irgb6 localization and percentage plaque loss on IFNγ-stimulated MEFs compared to unstimulated MEFs infected with the indicated strains. Strains that have greater than 90% mortality in CD-1 outbred mice [11] are indicated in red. The predicted reason for high IRG coating is indicated below the graph, which for CEP, VEG, CASTELLS and P89 is the unexpressed *ROP18_III_* allele, and for Pru, ME49, COUGAR and BOF is divergent or missing *ROP5* alleles. (B) Phylogenetic tree of *ROP5-A*, *B* and*C* and previously reported major and minor alleles [34] constructed from full-length coding nucleotide sequences using Neighbor-Joining with 1000 bootstraps. (C) Cumulative behavior, codon by codon, of synonymous (red), nonsynonymous (green) and insertion/deletion (black) mutations in *ROP5-A* (left) and *ROP5-B/C* (right). (D) Relative expression of *ROP5-A, ROP5-B/C* and *ROP18* determined by RNA-Seq of murine BMDM infected for 24 hours with the indicated strains. Samples were matched for similar levels of *Toxoplasma* RNA. *ROP5-A* and *B/C* copy number estimated by sequencing coverage of the *ROP5* locus versus the average genome coverage for strains for which the genome has been sequenced is indicated in the table below the graph.

Although mouse virulence has been determined for many non-canonical strains [11], it is unknown what factors determine virulence in these strains. We wondered if virulent non-canonical strains could also evade IRG-mediated killing, or if IRG evasion is specific to type I strains. We measured the percentage plaque loss in IFNγ-stimulated MEFs as well as percentage Irgb6-coated vacuoles for strains from haplogroups 1–11 [6], [41]. In general, IRG evasion correlates with virulence as strains that have a mortality rate of greater than 90% in CD-1 mice also have 25% or less Irgb6-coated vacuoles and plaque loss (Figure 3A). However, some exceptions are CASTELLS and COUGAR, which exhibit greater than 50% Irgb6 coating and plaque loss in IFNγ-stimulated MEFs, despite a high mortality rate in mice [11]. These strains may have a different mechanism underlying their virulence in mice besides IRG evasion.

For most strains, the Irgb6 coating and plaque loss correlates with their *ROP18* allele (Figures 3A and S2) [11]. For example, CASTELLS and P89, as well as the type III strains CEP and VEG, have between 40% and 50% Irgb6 coating, and all of these strains do not express ROP18 because they have a *ROP18_III_*-like allele that contains an insertion in the promoter [11]. The strains that express a type I-like allele of *ROP18*, with the exception of BOF, display 25% or less Irgb6 coating. Type II strains and COUGAR are highly susceptible to the IRGs with 70% and 53% Irgb6 coating respectively, despite having the virulent *ROP18_II_* allele. For type II strains, the avirulent *ROP5* locus likely explains the high degree of Irgb6 coating, but it is unknown what versions of *ROP5* are present in the non-canonical strains.

### ROP5 sequence and expression explain strain differences in IRG evasion

For most of the strains mentioned above, Irgb6 coating correlates with their *ROP18* allele, suggesting that they also have a virulent *ROP5* locus, as this is necessary for ROP18 to function (Figure 2). It is currently unknown what determines the virulence and IRG evasion properties of the *ROP5_I/III_* locus because both copy number and amino acid sequence of the individual copies differ between the canonical strains [33]. To identify differences that may be associated with virulence or IRG evasion, we sequenced the different *ROP5* isoforms of strains from haplogroups 1–11 (GenBank JQ743705-JQ743783). Based on the *Toxoplasma* genome sequence (www.ToxoDb.org) and our own genome sequencing of seven non-canonical *Toxoplasma* strains (Minot et al., submitted), we identified four distinct *ROP5* open reading frames that we amplified and sequenced separately using isoform specific primers. Sequence chromatograms indicated that two or more alleles were present for the second *ROP5* reading frame. We therefore cloned the PCR product from this *ROP5* gene and sequenced multiple clones to obtain sequences from the different alleles, but some alleles may still be missing. Sequences from this expanded paralog matched what has previously been called both *ROP5*-*B* (minor) and *C* (major) genes (Figure 3B) [33], [34]. We could not differentiate *B* and *C* alleles for all strains if they were not similar to the canonical strains, so we refer to them here as *B* copies. We determined that besides the three major *ROP5* copies that were previously described, 2 other highly divergent *ROP5* isoforms exist that we call *ROP5L-A* and *ROP5L*-*B* (Figures S3 and S4). Interestingly, *ROP5L-A* and *ROP5L*-*B* are highly conserved between strains, but we find that these isoforms are not expressed in tachyzoites (Figure S3) so they will not be discussed further. The previously described *ROP5* genes (*A, B* and *C*) [33] are highly divergent with strong evidence for diversifying selection (Figure 3C)., especially in surface exposed residues in the kinase domain [42]

In general, for *ROP5-A* and for *ROP5-B* and *C*, which cluster together, alleles can be divided into distinct groups with the BOF, P89, CAST and GPHT strains grouping with the virulent types I and III alleles (Figure 3B). A second allelic group consists of the strains VAND, RUB, GUY-KOE, GUY-DOS and GUY-MAT. The ability to confer virulence of this allelic group is unknown but because these strains are all highly virulent [11] and able to evade the IRGs, these alleles are likely virulent. A third very divergent group of alleles contains the strains MAS, CASTELLS and TgCatBr5, but there is less diversity in the *ROP5-A, B* and *C* isoforms present in these strains. The COUGAR allele is most similar to but divergent from the second group, but interestingly, COUGAR has only one *B/C* allele. The avirulent *ROP5* locus from type II is also divergent, and a phylogenetic analysis of all *ROP5* alleles indicates that the type II *ROP5-B* and *C* genes are more closely related to *ROP5-A* than to *ROP5-B* or *C* of the other strains. These results suggest that *ROP5-B* and/or *C* could be important for IRG evasion and virulence since type II strains and COUGAR have high levels of IRG coating (Figure 3A) and seem to have either ROP5 alleles that are all *ROP5-A*-like (type II) or are missing *ROP5-C* (COUGAR) (Figure 3B).

Next, we tested whether differences in ROP5 expression or copy number could account for strain differences in IRG evasion. For example, BOF has virulent *ROP18* and *ROP5* alleles but is highly coated by Irgb6 (Figure 3A–B). To estimate copy number differences between the strains we have sequenced, we plotted the sequencing coverage of the *ROP5* locus versus the average genome coverage, as this was previously shown to be a good estimate for copy number [43]. Most of the strains had about twice as many reads at *ROP5-A* and *B* as the rest of the genome, while MAS and TgCatBr5 have 3–5 copies of each gene (Figure 3D). However, coincident with our inability to amplify *ROP5-A*, we found that BOF is missing *ROP5-A* and has only one copy of *ROP5-B*.

**Figure 4:**
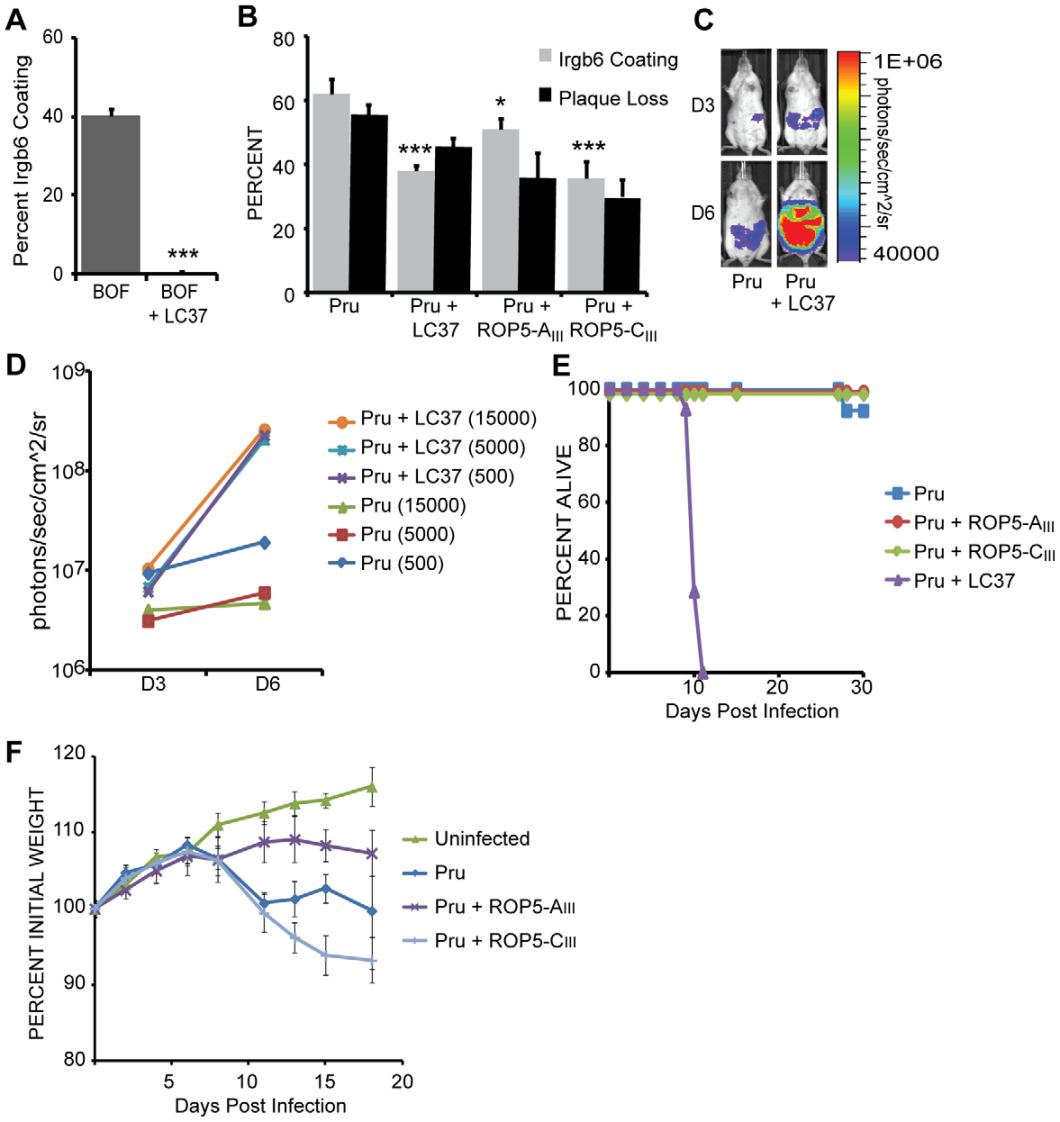
ROP5_III_-C, but not ROP5_III_A, inhibits IRG accumulation and increases mouse virulence. (A) Quantification of Irgb6 localization on the PV in IFNγ-stimulated MEFs infected with BOF and BOF + LC37. Mean + SEM, n = 3 experiments, ***p<0.001, Student's t-test. (B) Quantification of Irgb6 localization on the PV and percentage plaque loss on IFNγ-stimulated MEFs compared to unstimulated MEFs infected with Pru, Pru + LC37, Pru + ROP5_III_-A, and Pru + ROP5_III_-C. Mean + SEM, n>4 experiments, *p<0.05, ***p<0.001 One-way ANOVA with Bonferroni correction for all pairwise comparisons. (C) *In vivo* imaging of mice infected with firefly luciferase-expressing parasites of the indicated strains at days 3 and 6 post infection with 5000 parasites. One representative of 5 infected mice per strain is shown. (D) Quantification of *in vivo* imaging shown as average photons/sec/cm^2^/sr for infected mice at 3 doses on days 3 and 6. (E) Mouse survival after infection with indicated strains, n>8 for each strain, combined results for doses 500, 5000 and 15000 (Pru and Pru + LC37 only). (F) Average percent change in weight over time for mice infected with the indicated strains, Mean ± Std dev.

We also looked at ROP5 expression levels determined using RNA-Seq data from 24 hour infections of murine bone marrow derived macrophages with each strain (Figure 3D). BOF has barely detectable expression of *ROP5-B* and no expression of *ROP5-A*, likely explaining its high Irgb6 coating despite having a similar ROP5-B/C amino acid sequence to types I and III. Indeed BOF + LC37 has virtually no Irgb6 coating (0.33%) compared to BOF (40% Irgb6 coating, P = 0.001) (Figure 4A). *ROP5* expression levels can also likely explain many intra-haplogroup strain differences where *ROP18* and *ROP5* coding sequence are the same; for example, VEG has higher ROP5 expression levels compared to the other type III strain CEP, and VEG has slightly reduced IRG coating compared to CEP. Thus, higher ROP5 expression is correlated with reduced IRG coating, suggesting a non-enzymatic, dose-dependent role for ROP5 in IRG evasion.

### ROP5-C complements IRG evasion in type II parasites

Because the LC37 cosmid that reduced Irgb6 coating and plaque loss in S22 and BOF contains *ROP5-A*, *B* and *C* it is unknown which of these isoforms (or which combination) is important for IRG evasion. However, the fact that type II *ROP5* alleles are less divergent and more similar to *ROP5-A* suggests type II is missing *ROP5-B* and *C*. Additionally, *ROP5-C* was previously described as the major allele with *A* and *B* as minor alleles when trace reads were assembled for the ROP5 coding region of types I, II and III [34]. Therefore, we tested if *ROP5-A_III_*, *ROP5-C_III_* or LC37, which contains the entire *ROP5* locus, could complement IRG evasion in the type II background. Although some of the effects we see in the type II background will be due to an interaction with ROP18, because ROP18 is present in all backgrounds, we can still compare the effects of individual *ROP5* genes. We find, as expected, that expression of *ROP5-A_III_* in the type II strain Pru led to only a slight but significant reduction in Irgb6 coating (51%, P<0.05), but expression of *ROP5-C_III_* in Pru led to a significant reduction of IRG coating (36%, P<0.001) similar to that of Pru + LC37 (38%, P<0.001) compared to a heterologous control (62%) (Figure 4B). The 36% IRG coated vacuoles in Pru + ROP5-C_III_ is comparable to the 25% IRG coated vacuoles for GT1, suggesting that the lack of ROP5-C may account for the excessive IRG accumulation on type II vacuoles.

To see if ROP5-C_III_ can also increase the survival of type II parasites *in vivo*, we infected CD-1 mice with Pru, Pru + ROP5-A_III_, Pru + ROP5-C_III_ or Pru + LC37. The growth and dissemination of Pru and Pru + LC37 was determined by *in vivo* imaging of luciferase activity. On the third day post infection, Pru + LC37-infected mice had twice the parasite burden of Pru-infected mice (Figure 4C and D). By day six, there was 50 fold higher luciferase activity in Pru + LC37-infected mice (P<0.0001), and the parasites had disseminated throughout the peritoneal cavity. Indeed, 100% of Pru + LC37-infected mice died within 11 days of infection even at the lowest dose (Figure 4E). Mice infected with Pru parasites expressing only ROP5-A_III_ or ROP5-C_III_ survived the infection (Figure 4E) but Pru + ROP5-C_III_-infected mice had more ruffled fur and lost significantly more weight (Figure 4F) than Pru-infected mice throughout the course of infection(P = 0.01 at 15 days post infection) while Pru + ROP5-A_III_-infected mice continued to gain weight. Together, these results suggest that while expression of *ROP5-C_III_* can reduce Irgb6 coating of type II parasites, *ROP5-C_III_* only partially enhances the survival of type II parasites *in vivo*, and the whole *ROP5* locus is required to significantly increase virulence in mice.

### ROP5 does not interact with ROP18 and is not necessary for ROP18 kinase activity

**Figure 5:**
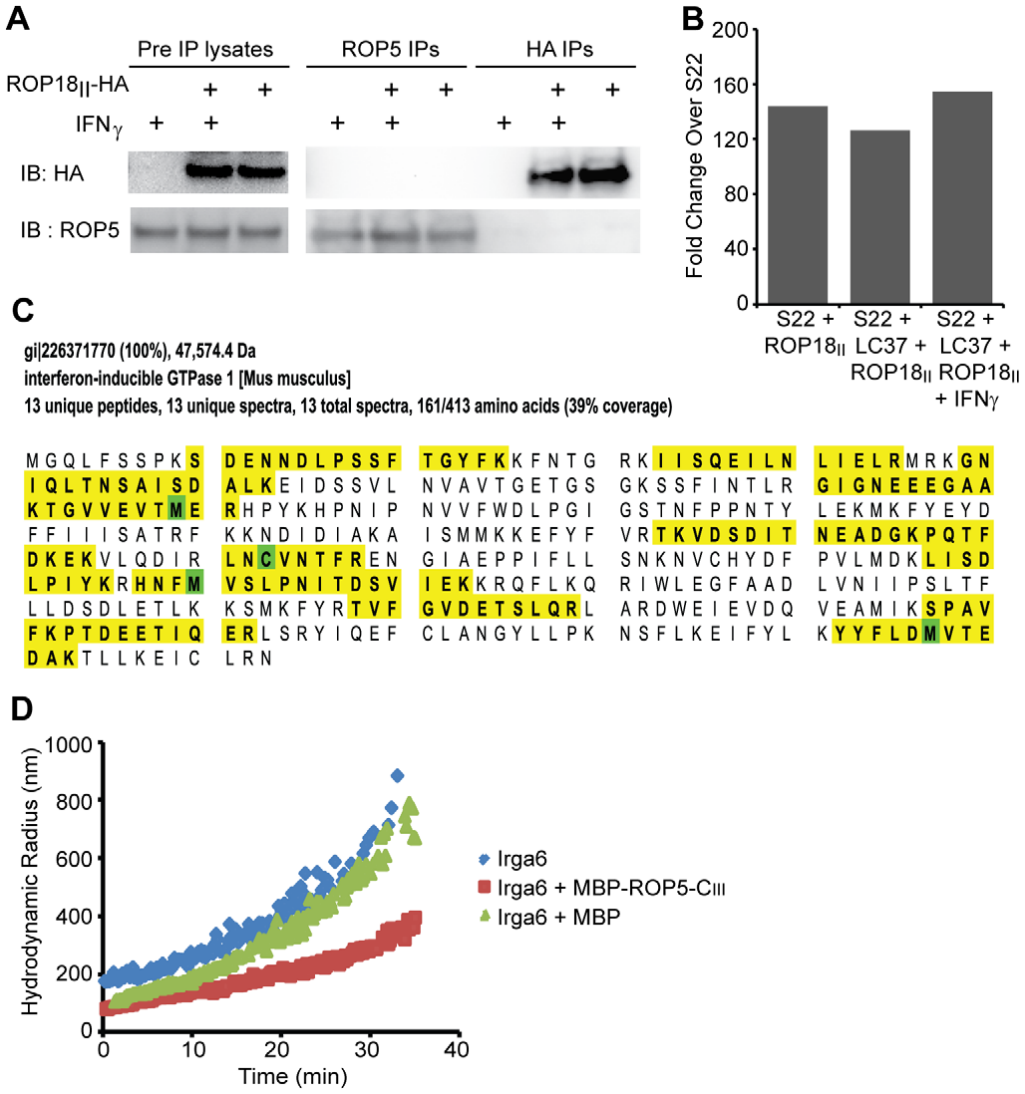
ROP5 interacts with Irga6, but not ROP18, and inhibits Irg6 oligomerization. (A) ROP5 and HA were immunoprecipitated from IFNγ-stimulated or unstimulated MEFs infected with CEP or CEP + ROP18_II_-HA and inputs and immunoprecipitates were Western blotted for ROP5 and HA. (B) Kinase activity of ROP18-HA immunoprecipitated from IFNγ-stimulated or unstimulated MEFs infected with S22, S22 + ROP18_II_-HA or S22 + LC37 + ROP18_II_-HA parasite strains. Half of the immunoprecipitated protein was Western blotted with anti-HA and the relative amount of ROP18-HA from each strain was quantified using the ImageQuant (GE Healthcare Life Sciences) software. The remaining immunoprecipitated proteins were incubated with ^32^P-γ-ATP and a model peptide substrate (Lim, D., submitted) and spotted in quadruplicate onto phospho-cellulose paper where the ^32^P-γ-ATP incorporation was quantified by phosphorimage analysis. The kinase activity is expressed as fold change over the S22 strain and normalized to the relative amounts of ROP18-HA that was immunoprecipitated. This experiment was performed twice and the graph represents the mean from those experiments. (C) Unique peptides (yellow) and percent sequence coverage of Irga6 recovered from mass spectrometry of proteins co-immunoprecipitated with ROP5-C_III_-HA. Briefly, HA-immunoprecipitated proteins from IFNγ-stimulated or unstimulated MEFs infected with Pru, Pru + ROP5-A_III_-HA, Pru + ROP5-C_III_-HA or RH + GRA15_II_-HA and lysed in the presence or absence of 0.5 mM GTPγS were separated by SDS-PAGE and analyzed by MS/MS. Irga6 peptides were recovered only in ROP5-C_III_-HA samples lysed in the presence of GTPγS. (D) Oligomerization of 20 µM Irga6 with10 mM GTP at 37°C in the presence of MBP-tagged ROP5-C or MBP shown as predicted mean hydrodynamic radius of the particle population determined by dynamic light scattering.

It is not clear how ROP5 inhibits IRG accumulation at the PVM, but other pseudokinases have been shown to serve as protein scaffolds or to regulate the activity of kinases [44]. Since ROP18 requires ROP5 for fully efficient IRG evasion, and there is an interactive effect of adding ROP18 and ROP5 to the S22 strain, it is possible that ROP5 and ROP18 interact directly. To test this hypothesis, we immunoprecipitated ROP5 and ROP18_II_-HA from MEFs infected with CEP or CEP + ROP18_II_-HA for one hour with or without previous IFNγ stimulation. We were unable to detect by western blot co-immunoprecipitation of ROP18 and ROP5 (Figure 5A). Furthermore, when recombinant, tagged ROP18 kinase domain (Lim et al., submitted) is added to cell lysates from IFNγ-stimulated or unstimulated MEFs infected for one hour with Pru + ROP5-C_III_HA, and ROP5 is immunoprecipitated with anti-HA, we do not co-immunoprecipitate ROP18 (Figure S5A) indicating that there is no direct interaction between ROP5-C_III_ and the ROP18 kinase domain. Next we tested the hypothesis that ROP18 is only active in the presence of virulent *ROP5* alleles by immunoprecipitating ROP18_II_-HA from MEFs infected with S22, S22 + ROP18_II_HA, and S22 + LC37 + ROP18_II_HA for one hour with or without previous IFNγ stimulation for use in an *in vitro* kinase assay. We found that there was no difference in the activity of ROP18 immunoprecipitated from parasites with or without a virulent ROP5, as measured by the phosphorylation of an optimized substrate (Lim et al., submitted) *in vitro*, (Figures 5B and S5B). This established that ROP18 was active in all backgrounds and indicated that there are no irreversible effects of ROP5 on ROP18 kinase activity.

### ROP5 directly interacts with and inhibits the oligomerization of Irga6

Because ROP5 does not directly interact with or irreversibly affect ROP18 kinase activity, we next tested the hypothesis that ROP5 directly interacts with one or more IRGs. We immunoprecipitated HA-tagged proteins from IFNγ-stimulated or untreated MEFs infected for one hour with Pru, Pru + ROP5-A_III_-HA, Pru + ROP5-C_III_-HA, or RH + GRA15_II_-HA and lysed in the presence or absence of GTPγS (a non-hydrolyzable form of GTP). Co-immunoprecipitated proteins were separated by SDS-PAGE and identified by mass-spectrometry. We did not recover any ROP18 peptides, again suggesting that ROP5 does not directly interact with ROP18. We did, however, recover 13 peptides (38% sequence coverage) from Irga6 only in the Pru + ROP5-C_III_-HA infected samples lysed in the presence of GTPγS (Figure 5C) suggesting a specific interaction between ROP5-C and Irga6 because the other HA-tagged, PVM associated proteins did not co-immunoprecipitate Irga6 under these conditions. Under different buffer conditions and in the absence of GTPγS, we also recovered 4 peptides of Irga6 and 2 peptides (9.8% sequence coverage) of Irgb10 only in the Pru + ROP5-C_III_-HA infected samples (data not shown). Because ROP5 lacks kinase activity [42] but reduces IRG localization to the PVM, we wondered if Irga6 binding by ROP5 could inhibit Irga6 oligomerization, which is necessary for its activity. To test this hypothesis, we measured the GTP-mediated oligomerization of recombinant Irga6 by dynamic light scattering in the presence of recombinant maltose binding protein (MBP)-tagged ROP5 or MBP alone. We found the predicted hydrodynamic radius of Irga6 to be reduced in the presence of ROP5 but not MBP (Figure 5D). Thus, we find that ROP5-C_III_ binds and inhibits the oligomerization of at least one IRG.

### ROP16 and GRA15 do not affect IRG evasion by Toxoplasma

**Figure 6:**
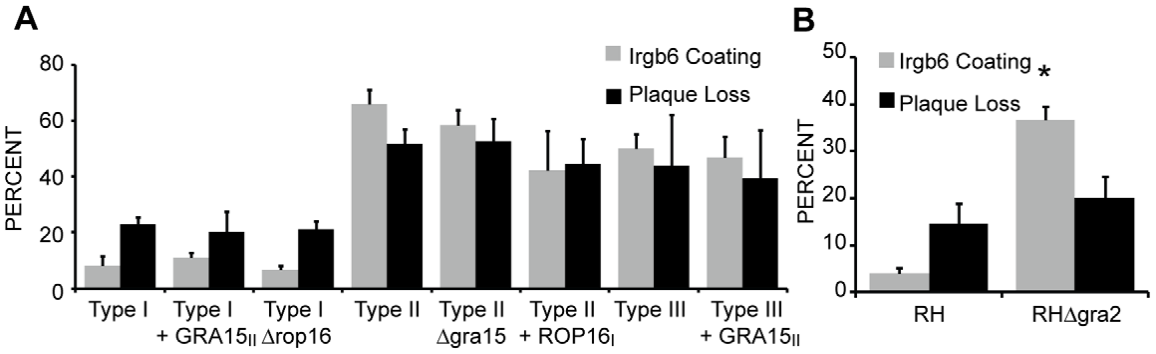
GRA2, but not ROP16 or GRA15, affects IRG evasion. WT MEFs stimulated for 24 hours with IFNγ and infected with the indicated strains for 1 hour and fixed for immunofluorescence or allowed to form plaques for 4–7 days. (A,B) Quantification of Irgb6 localization on the parasite containing vacuole and percent plaque loss on stimulated MEFs compared to unstimulated MEFs infected with the indicated strains. Mean + SEM, n>4 experiments. ***p<0.001, Student's t-test.

It was recently reported that p65 guanylate-binding proteins (GBPs), members of the dynamin superfamily that includes the IRGs, also accumulate on the *Toxoplasma* PVM alongside the IRGs [37]. Because ROP16 and GRA15 were shown to affect GBP coating, we were interested to see if ROP16 and GRA15 also affect IRG coating. We measured the effect of ROP16 and GRA15 on IRG coating and IRG-mediated killing in types I, II and III genetic backgrounds. In a type I background, the deletion of *ROP16*, the transgenic expression of *GRA15_II_*, or both in combination did not significantly alter IRG coating or killing (Figure 6A and not shown). Likewise, type IIΔ*gra15*, type II transgenically expressing *ROP16_I_*, and type III transgenically expressing *GRA15_II_* showed no statistical differences in Irgb6 coating or plaque loss compared to their parental strains. Thus, while these genes may affect GBP coating, they do not significantly alter Irgb6 accumulation.

### PVM structure affects IRG accumulation

Not all of the F1 progeny in the I×II cross that have the type I *ROP5* are as virulent as type I in mice [34] indicating that there are other genes besides *ROP5* and *ROP18* that affect virulence. While the genetic location of the dense granule protein GRA2 has not been verified as a QTL affecting virulence, an RHΔ*gra2* strain is one of the few type I knockouts that have reduced mouse virulence [45]. While the reason for this reduced virulence is unknown, it is known that GRA2 functions in the formation of the tubulovesicular network in the *Toxoplasma* PVM [46], which creates negative curvature in the PVM that might help to attract *Toxoplasma* proteins secreted into the host cell back to the PVM [47]. Indeed, it has been shown that the RHΔ*gra2* strain has reduced ROP18 localization to the tubulovesicular network in the *Toxoplasma* PVM [47]. We therefore hypothesized that this GRA2-dependent ROP18 and ROP5 localization and/or localization of other proteins, would be important for IRG evasion. Indeed, the RHΔ*gra2* strain has significantly increased IRG coating to 36% (P<0.001) and increased plaque loss on IFNγ-stimulated MEFs to 24% (P = 0.08) (Figure 6B). Therefore, a protein required for the formation of the PVM structure also affects IRG accumulation.

### Strain differences in survival in IFNγ-stimulated human foreskin fibroblasts

**Figure 7:**
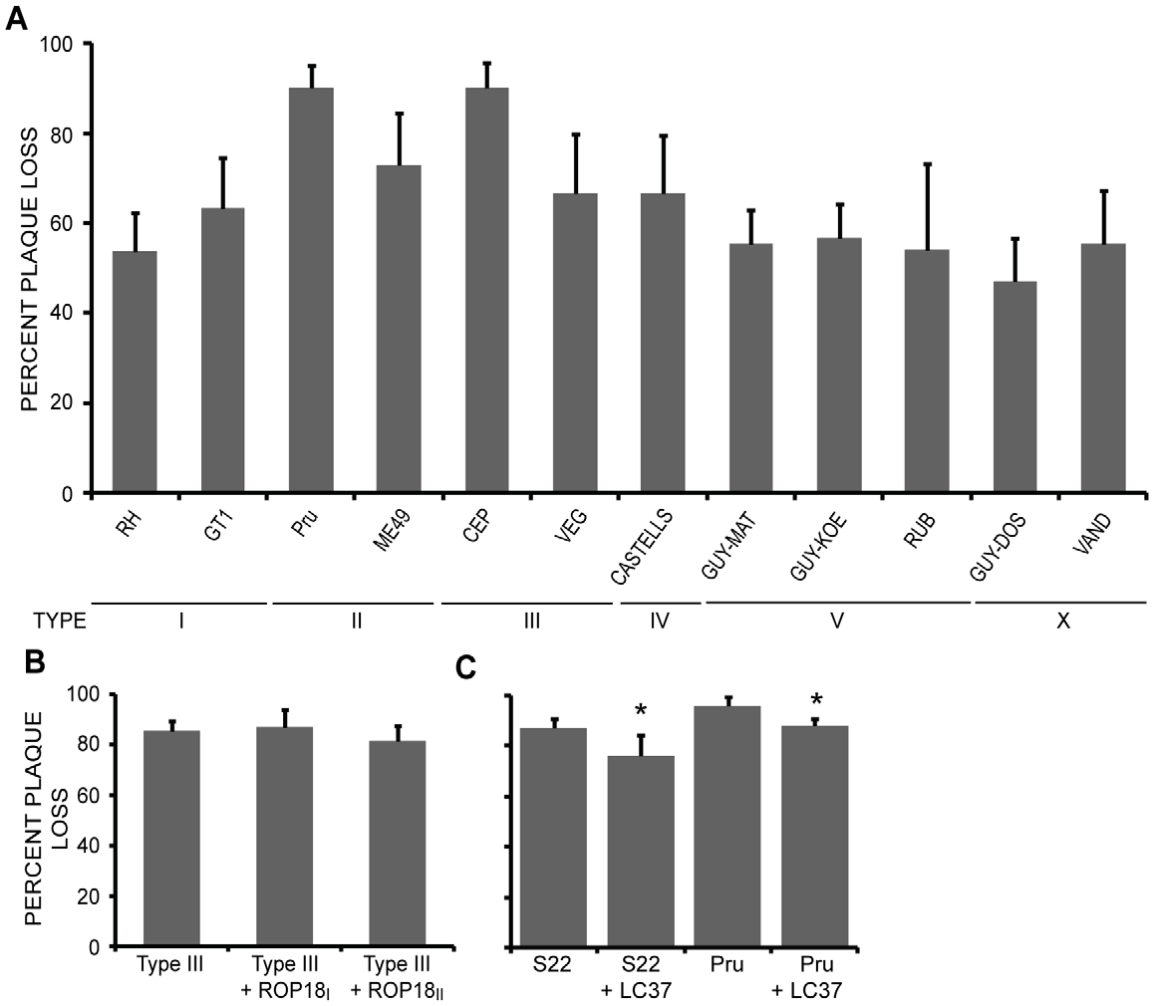
Strain differences in survival in IFNγ-stimulated human cells. Monolayers of HFFs, either previously stimulated for 24 hours with IFNγ or left untreated, were infected with 100–300 parasites. The number of plaques that form after 4–7 days of growth was determined. (A) Percent plaque loss of type I strains RH and GT1, type II strains ME49 and Pru, type III strains CEP and VEG, as well as the non-canonical strains CASTELLS, GUY-MAT, GUY-KOE, RUB, GUY-DOS and VAND. Mean + Std. dev., n≥3 experiments. (B) Percent plaque loss for type III, type III + ROP18_I_ and type III + ROP18_II_. Mean + Std. dev., n≥5 experiments. (C) Percent plaque loss of S22, S22 + LC37, Pru and Pru + LC37. Mean + Std. dev, n≥4 experiments, * p<0.05, Student's t-test.

We wondered if there are strain differences in the survival of *Toxoplasma* in IFNγ-stimulated human cells since strain differences in virulence have been primarily studied in mice, and human cells lack the multitude of IRGs present in murine cells. We measured the percentage plaque loss of different types I, II and III strains as well as of non-canonical strains in human foreskin fibroblasts (HFFs) pre-stimulated for 24 hours with IFNγ (Figure 7A). In general, the percentage plaque loss in IFNγ-stimulated HFFs is higher than in IFNγ-stimulated MEFs. The type I strains RH and GT1 have plaque losses of 54% and 63%, respectively, while the type II strains ME49 and Pru have plaque losses of 73% and 96%, respectively and the type III strains CEP and VEG have plaque losses of 90% and 67%, respectively. The non-canonical strains range in plaque loss from 47% (GUY-DOS) to 67% (CASTELLS). Thus, strain susceptibility to IFNγ-mediated killing in human cells does not correlate with that of murine cells.

### ROP18 and ROP5 have a minimal effect on IFNγ-mediated killing in human foreskin fibroblasts

As we have shown, ROP18 and ROP5 are responsible for most of the strain differences in IFNγ-susceptibility in murine cells, but *Toxoplasma* IFNγ-susceptibility in murine cells does not correlate with IFNγ-susceptibility in human cells. To test if ROP18 affects IFNγ-mediated killing in human cells, we first examined type III strains transgenically expressing a virulent copy of *ROP18*. Neither *ROP18_I_* nor *ROP18_II_* expression in type III decreases the percentage plaque loss compared to the parental strain (Figure 7B), suggesting that ROP18 is not responsible for strain differences in IFNγ-mediated killing in human cells. To see if ROP5 affects survival in IFNγ-activated human cells, we compared the percent plaque loss in IFNγ-stimulated HFFs between S22 and S22 + LC37 and between Pru and Pru + LC37. The plaque loss decreases from 87% for S22 to 76% for S22 + LC37 (P = 0.03) and from 96% in Pru to 88% for Pru + LC37 (P = 0.01) (Figure 7C). Although the differences in plaque loss due to expression of *ROP5* are significant, the differences are minimal (±10%). Thus, virulent alleles of *ROP18* and *ROP5* do not largely affect parasite survival in IFNγ-stimulated human cells.

## Discussion

We report that the precise allelic combination of the *Toxoplasma* polymorphic *ROP18* and *ROP5* genes determines *Toxoplasma* strain differences in susceptibility to killing by IFNγ-stimulated MEFs, even for non-canonical strains. We also show that ROP18 and ROP5 function by inhibiting the accumulation of and subsequent killing by the IRGs. *Toxoplasma* strains also differ in their susceptibility to killing by IFNγ-stimulated HFFs, but this is not determined by ROP18 or ROP5.

Previous studies on the role of ROP18 in mediating strain differences in IRG accumulation on the PVM have produced inconsistent results. Initial studies of *in vivo* primed macrophages infected with the type III strain CTG expressing ROP18_I_ and L929 cells expressing ROP18_I_ infected with the type II strain ME49 showed minimal effects of ROP18 on Irgb6 and Irga6 coating [19], [28]. More recently, ROP18_I_ was shown to phosphorylate a conserved threonine in the switch 1 loop of the GTPase domain of Irga6 and Irgb6 leading to their subsequent inactivation [30], [31]. Here, we report that both ROP18_I_ and ROP18_II_ can prevent the accumulation of IRGs on the PVM but only when expressed in a genetic background that contains the virulent *ROP5* locus. The lack of virulent ROP5 in type II strains therefore likely explains why L929 expression of ROP18_I_ did not affect IRG accumulation on type II vacuoles in those cells [19].

Previously it was shown that the avirulent strain S22 transgenic for the cosmid LC37, containing *ROP5*, had slightly fewer Irgb6 coated vacuoles (∼72%) than wild type S22 (87%) in IFNγ-stimulated MEFs, but growth inhibition as measured by uracil uptake was not affected [19]. In contrast, we see a significant decrease in the percentage of vacuoles coated with Irgb6 and increased parasite survival when comparing S22 + LC37 with S22. This could be due to the concentration of IFNγ, the exact assay used or the genotype of the host cells used, as the IRGs are divergent between mouse strains. We find that LC37 also reduces Irgb6 coating and promotes parasite survival in Pru and BOF, and that ROP5-C can explain most of the reduction in IRG coating *in vitro*. However, Pru + LC37 was significantly more virulent in mice than Pru + ROP5-C_III_ suggesting the other *ROP5* genes may have additional roles besides IRG evasion, in mouse virulence.

Currently, all *Toxoplasma* genes that determine strain differences in virulence were identified using pairwise crosses between types I, II and III. Because types I and III are progeny from a cross(es) between type II and a strain named alpha (similar to type VI) and beta (similar to type IX), respectively, these three strains are closely related to each other [48]. In recent years it has become appreciated that in South America, many other highly divergent strains exist, and types I, II and III are rarely isolated. To date, no studies have been done to determine the virulence determinants of these strains. Here we report that for these strains the allelic combination and/or expression level of *ROP18* and *ROP5* also determine how well these strains evade the accumulation of the IRGs and their virulence in mice. Surprisingly, even though the North American/European and South American strains diverged an estimated one million years ago [41], they all use the same two genes to evade the murine IFNγ response. This suggests that evasion of host IRGs is crucial for *Toxoplasma*. However, most strains do not completely evade the IRGs as this would be an unsuccessful strategy to ensure transmission in mice as the host would be killed before infectious cysts are formed. This could mean that *ROP18/ROP5* allelic combinations of highly virulent strains might have evolved to evade the IRGs of species that are more resistant to *Toxoplasma*, for example rats [49], and that mice are just an accidental host or it could be an artifact of the mouse lab strains commonly used. Strains such as type II, type III, BOF (VI), P89 (IX) and CASTELLS (IV) that either lack (type II) or do not express (BOF) virulent *ROP5* alleles or do not express *ROP18* (type III, P89 and CASTELLS) and are therefore less virulent in mice seem better adapted to cause chronic infections in mice. Indeed, the large majority of *Toxoplasma* isolates in North America and Europe belong to type II [2].

ROP5 reduces IRG coating of the *Toxoplasma* PVM independently of ROP18 despite a lack of kinase activity [42]. Many pseudokinases have been shown to act as scaffolds or regulators of active kinases [44]. We find that ROP5 is not necessary for ROP18 kinase activity *in vitro* nor did we find evidence for any direct interactions between ROP5 and ROP18 (Figure 5A). We find instead that ROP5 directly interacts with and inhibits the oligomerization of Irga6 (Figure 5C and D). Expression levels of ROP5 seem to correlate with the intra-haplotype differences in IRG coating between CEP and VEG, supporting a non-enzymatic, dose-dependent inhibition of the IRGs by ROP5. Importantly, both the IRGs and the *ROP5* locus have expanded, perhaps due to an evolutionary arms race whereby new host IRG genes required new *ROP5* genes so *Toxoplasma* could continue to evade IFNγ-mediated killing. Although ROP5 can inhibit IRG oligomerization, we see an interactive effect with ROP18 on IRG-coating and virulence. Perhaps the reduced oligomerization of IRGs in the presence of virulent *ROP5* alleles is reversible, but this de-oligomerization might provide access for ROP18 to bind and phosphorylate the IRGs on the threonines in their switch I loop, to prevent re-activation. If this model is correct than the interaction of ROP18 with the IRGs [30], [31] should only occur in the presence of virulent ROP5 alleles.

To defend itself against the IRGs *Toxoplasma* must have evolved a mechanism to ensure appropriate trafficking of ROP18 and ROP5 to the PVM upon their secretion into the host cytoplasm. The N-terminal amphipathic helices (RAH domains) of both proteins are required for efficient localization to the PVM, and it was speculated that their specificity for the PVM versus other membranes might be because of a preference for negative curvature [47]. Indeed, we found that RHΔ*gra2* parasites that have a disrupted tubulovesicular network [46], which provides much of the negative curvature of the PVM, have increased IRG accumulation. This indicates that the attraction of ROP5, ROP18 and possibly other secreted proteins to the PVM, which is attenuated in RHΔ*gra2* [47], outweighs the possible attraction the IRGs may have for the negative curvature of the PVM [50]. It is likely that the increased IRG accumulation on the PVM of RHΔ*gra2* accounts for its decrease in virulence [45].

Because all *Toxoplasma* strains seem to rely on ROP5 and ROP18 for evasion of the murine IFNγ response, these proteins could be attractive drug targets if they are also involved in evasion of the human IFNγ response. However, we find that although there are significant strain differences in susceptibility to IFNγ-mediated killing by HFFs, ROP5 and ROP18 do not markedly affect survival in those cells. This might not be surprising because humans do not possess the large variety of IRGs of murine cells (23 members) but only a single member (IRGM) that is not regulated by IFNγ [22]. The effector mechanisms induced by IFNγ in human cells that are effective against *Toxoplasma* include tryptophan degradation [25], iron depletion [51], P2X_7_-mediated death of the host cell [52] and activation of the NALP1 inflammasome [27]. While the IRGs do not mediate vacuolar destruction in human cells, we wondered if another group of dynamin-related large GTPase, the GBPs, could be involved in IFNγ-mediated killing by HFFs, but we failed to see GBP1 at the PVM in HFFs (data not shown).

The *Toxoplasma* strains that were most resistant to IFNγ-mediated killing by HFFs have also been shown to be able to cause severe disease even in immunocompetent humans. In future studies, strain differences in survival in IFNγ-activated HFFs may provide insight into that mechanism.

## Materials and Methods

### Reagents

A rat monoclonal antibody against HA (3F10, Roche, 1∶500 dilution), a goat polyclonal antibody against mouse TGTP (A-20, Santa Cruz Biotechnology, 1∶100 dilution), a mouse monoclonal antibody against *Toxoplasma* surface antigen (SAG)-1 (DG52) [53], and a mouse polyclonal antibody against the N-terminus of ROP5 [54] were used in immunofluorescence assays or immunoprecipitations. Secondary antibodies for immunofluorescence were coupled with Alexa Fluor 488 or Alexa Fluor 594 (Molecular Probes). Secondary antibodies used in Western blotting were conjugated to peroxidase (Kirkegaard & Perry Laboratories). Mouse IFNγ from Peprotech and human IFNγ from AbD serotec were dissolved in DMEM with 10% FBS.

### Parasites and cells

Parasites were maintained *in vitro* by serial passage on monolayers of human foreskin fibroblasts (HFFs) at 37°C in 5% CO2. The following representatives for each haplotype were used: RH and GT1 for type I, ME49 and Pru for type II, CEP and VEG for type III, MAS and CASTELLS for type IV, GUY-KOE and GUY-MAT for type V, GPHT and BOF for type VI, CAST for type VII, TgCatBr5 for type VIII, P89 for type IX, GUY-DOS and VAND for type X and COUGAR for type XI. A Pru strain engineered to express firefly luciferase and GFP (PruΔHXGPRT A7) [55], a CEP and RH strain engineered to express clickbeetle luciferase and GFP (CEPΔHXGPRT C22 and RH 1-1) [56], CEP + ROP18_II_, Pru + ROP16_I_ [7], RHΔ*gra2* [45], RHΔ*rop16* [57], RH + GRA15_II_ and CEP + GRA15_II_ [36] have been described previously. HFFs were grown as described previously [36]. WT C57BL6/J MEFs were a gift from A. Sinai (University of Kentucky College of Medicine, Lexington, KY), Atg7+/− and Atg7−/− MEFs [58] from Masaaki Komatsu (The Tokyo Metropolitan Institute Medical Science) and all MEFs were grown in HFF media supplemented with 10 mM Hepes. All parasite strains and cell lines were routinely checked for Mycoplasma contamination and it was never detected.

### Immunofluorescence assays

Monolayers of MEF cells grown on coverslips and incubated for 24 hours with or without 1000 U/ml IFNγ. Parasites were allowed to invade for 20 minutes, non-invading parasites were then washed away with PBS 3 times, and the infection proceeded for 1 hour. The cells were then fixed with 3% (v/v) formaldehyde in PBS for 20 minutes at room temperature, permeabilized with 0.2% saponin and blocked in PBS with 3% (w/v) BSA and 5% (v/v) FBS. Percent Irgb6 coating was determined in a blind fashion by finding intracellular parasites and then scoring Irgb6 coating as positive or negative.

### Characterization of ROP5 sequences

The coding sequence for ROP5 from types I (GT1), II (ME49), and III (VEG) was predicted from ToxoDB genomic sequence using ORF Finder (NCBI). ROP5 genomic DNA from additional strains was amplified by PCR with isoform specific primers confirmed by sequence chromatograms. *ROP5* was amplified with the following primers forward 5′CGATTCACGCTTTCCATGT′3, reverse 5′TCCTTCAGCGGAAAACAGAT′3 for ROP5-A, forward 5′CATTTCATGCCTTCCCAGTT′3, reverse 5′GCGCTCGAGTACTTGTCCTG′3 for ROP5-B/C, forward 5′GTCCCTGGAAAACTGTTTCG′3, reverse 5′GTGAACAGAGAGCGTCCAA′3 for ROP5-D, forward 5′ATTCTGCAATGCCCAAAAGA′3, reverse 5′TTCATGTTGGATACGGCAAC′3 for ROP5-E and 5′AAAAGGCGCGGCGAGCTAGCGTC′3 as an alternate forward primer for ROP5-A for MAS and CASTELLS.

The ROP5-B/C PCR products produced mixed sequence and therefore the PCR product was cloned and multiple clones were sequenced.

The following primers were used to sequence ROP5-A and ROP5-B 5′ATAGGTAACCGGGACGCTTG′3, 5′CCACTTCGGAAGAGACTTGC′3, 5′GGACAGACGCAGGCTTTTAC′3

The following primers were used to sequence ROP5-D and ROP5-E 5′TGAGCTGAAAACCGACTTCAC′3, 5′GGTGACTGGAACACTCGACA′3, 5′TTTTCCGGACCTTGTCTTTG′3, 5′TTCGGGAGAGACTTGCTCAG′3, 5′GCTGTGACAGTTCCGACTCA′3

Sequences were aligned using ClustalX and Neighbor-Joining phylogenetic trees were made with Molecular Evolutionary Genetic Analysis (MEGA) software version 4.1 with 1000 bootstraps and default settings [59]. The Non-synonymous Analysis Program (SNAP) was used to calculate the proportion of synonymous and non-synonymous changes in coding regions [60].

### Generation of transgenic parasites

The coding region and putative promoter *(766* bp upstream of the start codon for *ROP5-A* and 681 bp upstream for *ROP5-B)* of ROP5-A and ROP5-B was amplified from type III *Toxoplasma* genomic DNA by PCR *(A forward,* *5′-*CCACGCATTCTTCCACTCAGTACCG*-3′* *; B forward,* *5′-*CCACAATGGCTACCAGGTCCTGCG*-3′* *; A/B reverse,* *5′-*CTA*CGCGTAGTCCGGGACGTCGTA*CGGGTAAGCGACTGAGGGCGC*-3′* *).* The coding region of ROP18, along with putative promoter (742 bp upstream of the ATG start codon), from type I *Toxoplasma* genomic DNA was amplified by PCR. (Forward 5′-CACCAGATTCGAAACGCGGAAGTA-3′; Reverse 5′-TTACGCGTAGTCCGGGACGTCGTACGGGTATTCTGTGTGGAGATGTTCCTGCTGTTC -3′). These primers amplified these genes specifically as confirmed by sequencing and the sequence matched the previously published data [30]–[34]. Sequence coding for an HA tag was included in the reverse primer (denoted with italics) to C-terminally tag the protein. ROP5-A_III_HA, ROP5-C_III_HA and ROP18_I_ were then cloned into pENTR/D-TOPO (Invitrogen), and then cloned into pTKO-att (described in [58]) by LR recombination (Invitrogen). The pTKO-att-ROP5_III_HA vectors were then linearized by digestion with HindIII (NEB), which does not cut inside either gene. Linearized vector was transfected into PruΔHXGPRT parasites by electroporation as described previously [58]. The pTKO-att-ROP18 vector was linearized by digestion with NdeI (NEB) and transfected into CEPΔHXGPRT C22 parasites by electroporation. Stable integrants were selected in media with 25 mg/ml mycophenolic acid (Axxora) and 25 mg/ml xanthine (Alfa Aesar) and cloned by limiting dilution. To express ROP18_II_ in the S22 and S22 LC37 parasite strains, 35 µg of pTKO-att-ROP18_II_HA [1] was linearized by HindIII (NEB) and 1 µg of pTUB-CAT were co-transfected by electroporation. Stable integrants were selected by passage of 10^6^ parasites every 2 days in *2* µM chloramphenicol. Expression of ROP18 and ROP5_III_ was confirmed by IF and Western blot for HA staining (Figure 6A–D). The LC37 cosmid from the pSC/Ble library (gift of M.J. Gubbels, Boston College, Boston, MA) was expressed in PruΔHXGPRT A7 and BOF by transfecting 50 µg cosmid and selecting twice extracellularly for 1.5 hours with 5 µg/ml phleomycin. Integration was confirmed by PCR with the Type I ROP5 specific forward primer (5′-TTTTCCGCAGGCCGTGGC-3′) and ROP5A/B reverse for Pru and amplification of ROP5-A for BOF.

### Plaque assays

For the plaque assays, 100–300 parasites per well were added to monolayers of MEFs seeded the day before or HFFs seeded two days before and either previously stimulated with 1000 U/ml mouse IFNγ, 100 U/ml human IFNγ or left unstimulated for 24 hours before infection in a 24 well plate in either MEF media or DMEM with 1% FBS for HFFs. Infections were then incubated for 4–7 days at 37°C and the number of plaques was counted using a microscope.

### In vivo imaging analysis

CD-1 (Charles River Laboratories) mice were intraperitoneally (i.p.) infected with 500 or 5000 syringe-lysed tachyzoites in 300 µl PBS using a 28 gauge needle. On days 3, 6 and 12 post infection, parasite burden and dissemination was measured by bioluminescence emission imaging. Mice were injected i.p. with 3 mg firefly D-luciferin (Gold Biotechnology) dissolved in PBS, anesthetized with isofluorane, and imaged with an IVIS Spectrum-bioluminescent and fluorescent imaging system (Xenogen Corporation). Images were processed and analyzed with Living Image software. The MIT Committee on Animal Care approved all protocols. All mice were maintained in specific pathogen-free conditions, in accordance with institutional and federal regulations.

### High-throughput genomic and RNA sequencing

For genomic sequencing, DNA was isolated from freshly lysed *Toxoplasma* parasites using a Trizol-based extraction (Invitrogen). This DNA was subsequently prepared for high-throughput sequencing according to the Illumina single-end genomic DNA kit protocol (COUGAR, CASTELLS and MAS) and 36 nucleotides of each library was sequenced on an Illumina GAII and processed using the standard Illumina pipeline. Paired-end sequencing Illumina libraries were constructed for the genomic DNA of P89, GUY-KOE, TgCatBr5, BOF using the Nextera Illumina compatible DNA sample prep kit (Epicenter) and amplified with the modified PCR protocols described previously [61]. Sequence reads were aligned to the *Toxoplasma* and human genomes using the Maq software package [62]. Reference *Toxoplasma* genomes from a type II (Me49), a type I (GT1) and a type III (VEG) strain were obtained from http://toxodb.org (release 6.3). For RNA sequencing, murine bone-marrow derived macrophages (BMDM) were seeded in 6 well plates at 70% confluency and infected with different strains of *Toxoplasma* at three multiplicity of infections (MOIs): 15, 10 and 7.5. After 24 hours total RNA was extracted from all infected cells using the Qiagen RNeasy Plus kit. Integrity, size and concentration of RNA was then checked using the Agilent 2100 Bioanalyzer. The RNA was processed for high-throughput sequencing according to standard Illumina protocols. Briefly, after mRNA pull down from total RNA using Dynabeads mRNA Purification Kit (Invitrogen), mRNA was fragmented into 200-400 base pair-long fragments and reverse transcribed to into cDNA, before Illumina sequencing adapters were added to each end. Libraries were barcoded and subject to paired end sequencing on the Illumina HiSeq2000 (40+40 nucleotides) and processed using the standard Illumina pipeline. All libraries were spiked with trace amounts of the phiX bacteriophage for quality control purposes. After sequencing, the samples were de-barcoded to separate reads from the multiplexed samples using a custom Perl script. Reads were assembled into full sequences by mapping to exons and across exon junctions using the organism's genomes as a template. Maq was used to estimate *Toxoplasma* transcript abundance for ROP5 and ROP18 based on our sequenced alleles. A more detailed analysis of the genome and RNA-seq data will be described elsewhere.

### Immunoprecipitations, Western blotting and kinase assays

Immunoprecipitations were each performed with 5 µg of rat anti-HA (3F10, Roche) or mouse anti-ROP5 [54] conjugated to 25 µl of protein G dynabead slurry (Life Technologies). The HA antibodies were crosslinked at room temperature with 5 mM Bis(Sulfosuccinimidyl) suberate (*BS3*) (Pierce) prepared in conjugation buffer (20 mM sodium phosophate, 150 mM sodium chloride, pH 7.5) for 30 minutes and quenched by adding 50 mM Tris-Cl, pH 7.5 for 15 minutes and finally washed with conjugation buffer. For each immunoprecipitation (IP) condition, 4.2×10^6^ MEFs were infected at an MOI of ∼5–10, with the strain and condition indicated. After 1 hour, uninvaded parasites were washed away with PBS and the infected cells were treated with 0.25% trypsin for 5 minutes at 37°C. The cells were quenched and harvested with growth media and subsequently washed with PBS + 1 mM PMSF and lysed for 15 minutes at 4°C with light agitation in 1 ml of IP lysis buffer [50 mM HEPES-KOH (pH 7.5), 300 mM NaCl, 10 mM β-glycerophosphate, 1 mM NaF, 0.1 mM NaVO_4_, 1 mM PMSF, 1% NP-40, and protease inhibitor cocktail (Roche)]. The lysate was then centrifuged at 16,000 g for 30 minutes at 4°C and the supernatant was collected. For ROP18 binding assays, 1 µg of ROP18 recombinant kinase domain [residues 187–554, fused to a series of N-terminal fusion tags consisting of: (His_6_)-(glutathione S-transferase)-(maltose binding protein)-(*Streptococcus* protein B1 domain)-(TEV cleavage site), (Lim, D et al., submitted)] were incubated for 30 minutes before adding conjugated and crosslinked antibody beads described above and agitating them for 3 hours at 4°C. The beads were washed 3 times with IP wash buffer [10 mM HEPES-KOH (pH 7.5), 150 mM NaCl, 20 mM β-glycerophosphate, and 0.5% NP-40], washed 3× with HEPES-buffered saline (HBS) and boiled in sample buffer. The samples were western blotted with anti-GST HRP conjugate (GE Healthcare Life Sciences) and anti-HA (3F10, Roche) antibodies.

Immunoprecipitations for kinase assays were performed as above but with several changes. The cleared lysates were incubated with 10 µg of rat anti-HA (3F10, Roche) per IP reaction and incubated for 90 minutes, washed 5 times with the IP lysis buffer and 3 times with IP wash buffers and HBS (all buffers contained 300 mM NaCl). Half of the beads were boiled in sample buffer for western blotting with anti-HA and the other half used for the kinase assay. Kinase assays using a ROP18 model peptide substrate (NH_3_-KKKKKWISEHTRYFF-CONH_2_) (Lim, D. et al., submitted) were conducted at room temperature with a reaction buffer consisting of 50 mM HEPES pH 7.5, 300 mM NaCl, 10 mM DTT, 10 mM MgSO_4_ and 60 µM cold ATP. Each reaction contained 0.5 mM of peptide substrate and 2 to 10 µCi of ^32^P-γ-ATP. Reactions were stopped after 30 minutes by spotting on Whatman P81 phospho-cellulose paper, which were then dried and washed with 0.425% phosphoric acid until no significant radioactivity remained in the washes. Radioactivity captured on P81 filters was then quantified by phosphorimage analysis with ImageQuant 5.2 software (Molecular Dynamics). The radioactivity detected was normalized to the amount of protein immunoprecipitated as determined by the above Western blot.

### Mass spectrometry

Immunoprecipitations were performed as above with a monolayer of confluent MEFs in a T175 lysed in the presence or absence of 0.5 mM GTPγS (Sigma) and precipitated using 30 µg of HA antibody. The washed beads were boiled in sample buffer and samples were subjected to SDS–PAGE and colloidal coomassie (Invitrogen) staining. For mass spectrometry analysis, proteins were excised from each lane of a coomassie-stained SDS-PAGE gel encompassing the entire molecular weight range. Trypsin digested extracts were analyzed by reversed phase HPLC and a ThermoFisher LTQ linear ion trap mass spectrometer. Peptides were identified from the MS data using SEQUEST algorithms44 that searched a species-specific database generated from NCBI's non-redundant (nr.fasta) database.

### Dynamic light scattering

Recombinant Irga6 [residues 1–413, fused to a series of N-terminal fusion tags consisting of: (His_6_)-(glutathione S-transferase)-(maltose binding protein)-(*Streptococcus* protein B1 domain)-(TEV cleavage site), (Lim et al, submitted)] oligomerization was monitored in 50 mM Tris/5 mM MgCl_2_/2 mM DTT by dynamic light scattering (DLS). Oligomerization was initiated by the addition of 10 mM GTP (Sigma) to 20 µM Irga6 in the presence or absence of 40 µM recombinant (His_6_)-MBP-tagged ROP5-C_III_ or (His_6_)-MBP. The reaction was mixed by pipetting and immediately transferred to a quartz cuvette and equilibrated to 37°C. DLS was performed using a DynaPro NanoStar Light Scatterer (Wyatt Technologies) with an acquisition time of 10 sec over 35 minutes and analyzed using the DYNAMICS software version 7.1.4. The mean hydrodynamic radius of the population was estimated using the standard curve of molecular weight for globular proteins and is not equal to the actual size of the oligomer.

### Ethics statement

This study was carried out in strict accordance with the recommendations in the Guide for the Care and Use of Laboratory Animals of the National Institutes of Health. The MIT Committee on Animal Care (assurance number A-3125-01) approved all protocols. All mice were maintained in specific pathogen-free conditions, and all efforts were made to minimize suffering.

### Accession numbers

Sequences can be accessed in GenBank: ROP5-BL sequences JQ743705–JQ743719, ROP5-AL JQ743720–JQ743735, ROP5A–C sequences JQ743736–JQ743783.

## Supporting Information

Figure S1 **IFNγ-induced plaque loss is reduced when the IRGs are mis-regulated.** Monolayers of Atg7+/− and Atg7−/− MEFs were stimulated for 24 hours with IFNγ and infected with type II (Pru) for 1 hour or allowed to form plaques for 7 days. Immunofluorescence of Irgb6 PV coating and percent plaque loss on stimulated compared to unstimulated MEFs. Mean + SEM, n = 3 experiments, *P<0.05, ***P<0.001, Student's t-test.
(TIF)

Figure S2 **Phylogenetic analysis of ROP18.** Phylogenetic analysis of coding nucleotide sequences by Neighbor Joining with 1000 bootstraps of ROP18 alleles [11].
(TIF)

Figure S3 **Phylogenetic analysis and expression of ROP5L-A and B.** (A) ROP5L-A and B (B) phylogenetic analysis of coding nucleotide sequences by Neighbor Joining and cumulative mutations codon by codon by type (C and D respectively). E) Expression by RNA-Seq analysis of 24 hour infection with indicated strains in bone marrow-derived macrophages.
(TIF)

Figure S4 **Amino acid alignments of sequenced ROP5 genes.** Amino acid alignments of ROP5-A and B/C as well as ROP5L-A and B for sequenced strains.
(PDF)

Figure S5 **ROP5 does not directly interact with ROP18 and is not necessary for ROP18 kinase activity.** (A) Wild-type (wt) or mutant R223E recombinant proteins comprising the kinase domain (KD) of ROP18_I_ fused to MBP-GST were added to lysates prepared from IFNγ-stimulated and unstimulated MEFs infected with type II or type II + ROP5C_III_-HA parasites and incubated for 30 minutes before immunoprecipitating the reactions with anti-HA. Both the immunoprecipitates (lanes 1–6) and pre-IP lysates (lanes 7–12) were Western blotted with anti-GST and anti-HA. (B) Kinase activity of ROP18-HA immunoprecipitated from IFNγ-stimulated or unstimulated MEFs infected with S22, S22 + ROP18_II_-HA or S22 + LC37 + ROP18_II_-HA parasite strains. Half of the immunoprecipitated protein was Western blotted with anti-HA (top). The remaining immunoprecipitated proteins were incubated with ^32^P-γ-ATP and a model peptide substrate (Lim, D., submitted) and spotted in quadruplicate onto phospho-cellulose paper where the ^32^P-γ-ATP incorporation was quantified by phosphorimage analysis (bottom). This experiment was performed twice and the graph represents the mean from those experiments.
(TIF)

Figure S6 **Expression and localization of transgenic ROP18 and ROP5.** (A) Immunofluorescence of HA (red) in S22 + ROP18_II_-HA and S22 + LC37 + ROP18_II_-HA parasites as well as DIC and merged images. (B) Western blot for HA (top) and SAG1 (bottom) comparing expression of S22 + ROP18_II_-HA and S22 + LC37 + ROP18_II_-HA strains used for mouse infections. (C) Immunofluorescence of HA (red) in Pru + ROP5-A_III_-HA and Pru + ROP5-C_III_-HA parasites as well as DIC and merged images. (D) Western blot for HA (top) and SAG1 (bottom) comparing expression of Pru + ROP5-A_III_-HA and Pru + ROP5-C_III_-HA strains used for mouse infections.
(TIF)
