# Supplementary figures and images for: The Rhoptry Proteins ROP18 and ROP5 Mediate Toxoplasma gondii Evasion of the Murine, But Not the Human, Interferon-Gamma Response
Source: PLoS Pathog. 2012 Jun 28;8(6):e1002784. doi: 10.1371/journal.ppat.1002784 (PMC3386190; doi:10.1371/journal.ppat.1002784)

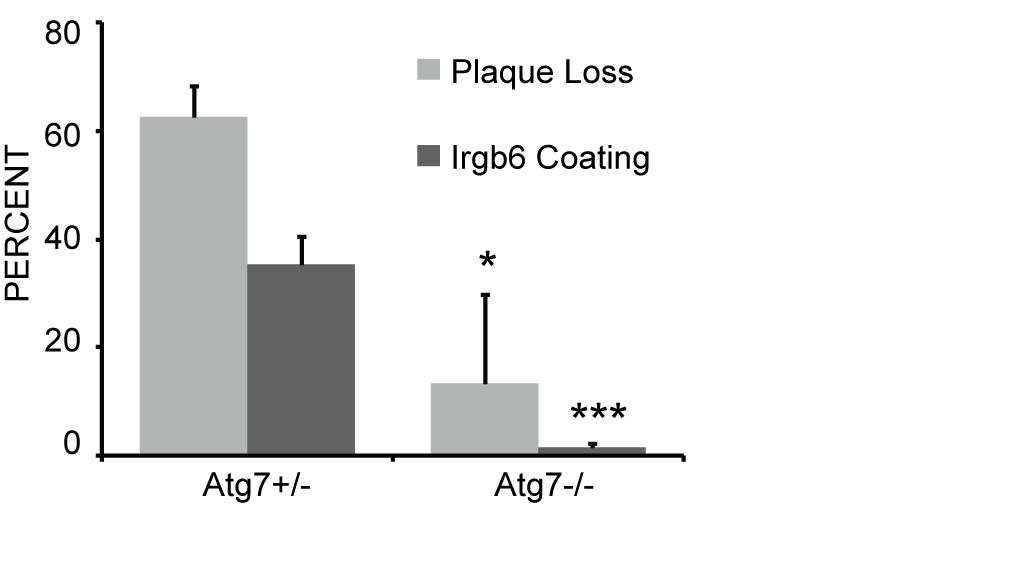

Supplement: Figure S1 — IFNγ-induced plaque loss is reduced when the IRGs are mis-regulated. Monolayers of Atg7+/− and Atg7−/− MEFs were stimulated for 24 hours with IFNγ and infected with type II (Pru) for 1 hour or allowed to form plaques for 7 days. Immunofluorescence of Irgb6 PV coating and percent plaque loss on stimulated compared to unstimulated MEFs. Mean + SEM, n = 3 experiments, *P<0.05, ***P<0.001, Student's t-test. (TIF) [file ppat.1002784.s001.tif]

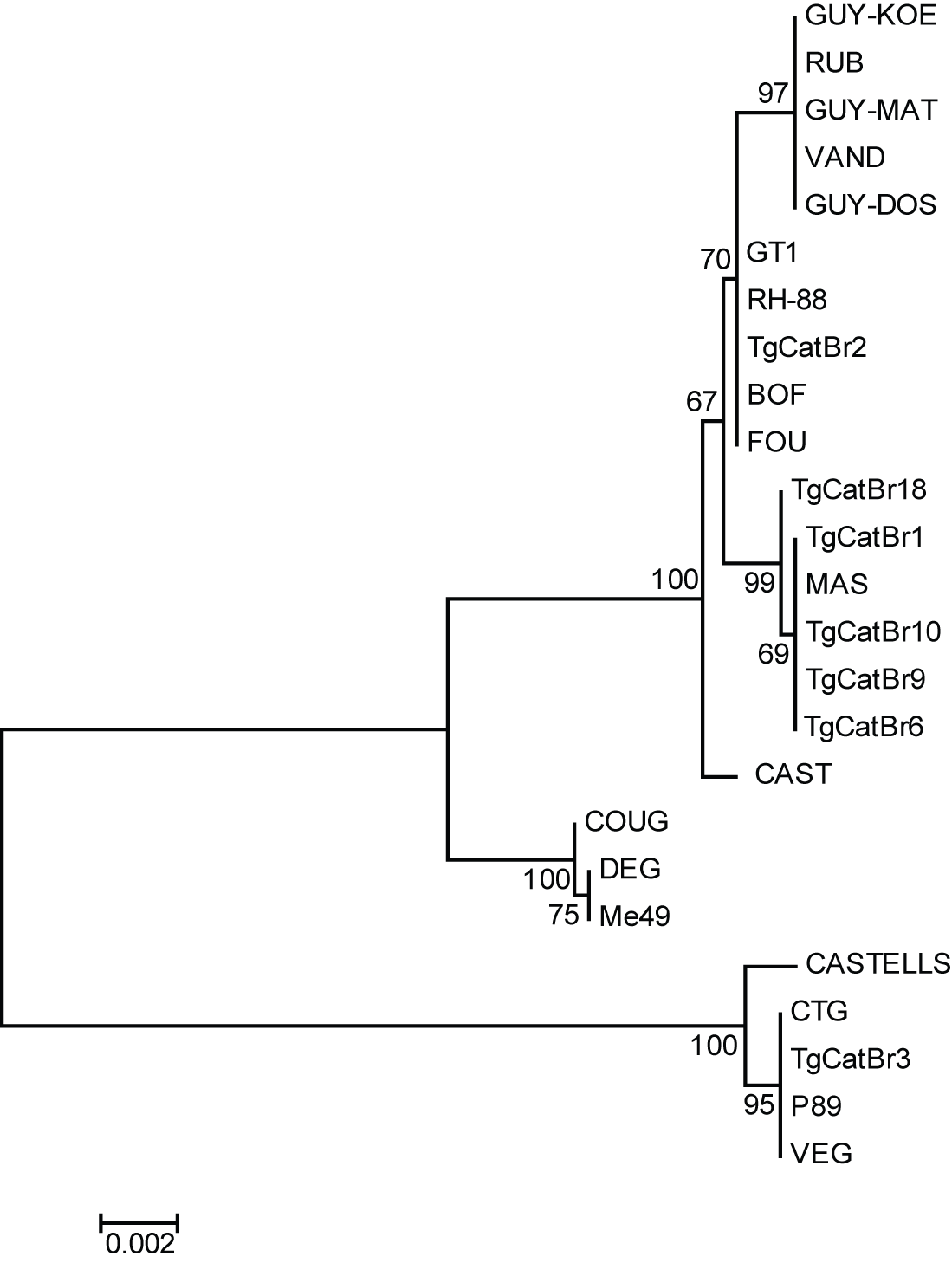

Supplement: Figure S2 — Phylogenetic analysis of ROP18. Phylogenetic analysis of coding nucleotide sequences by Neighbor Joining with 1000 bootstraps of ROP18 alleles [11]. (TIF) [file ppat.1002784.s002.tif]

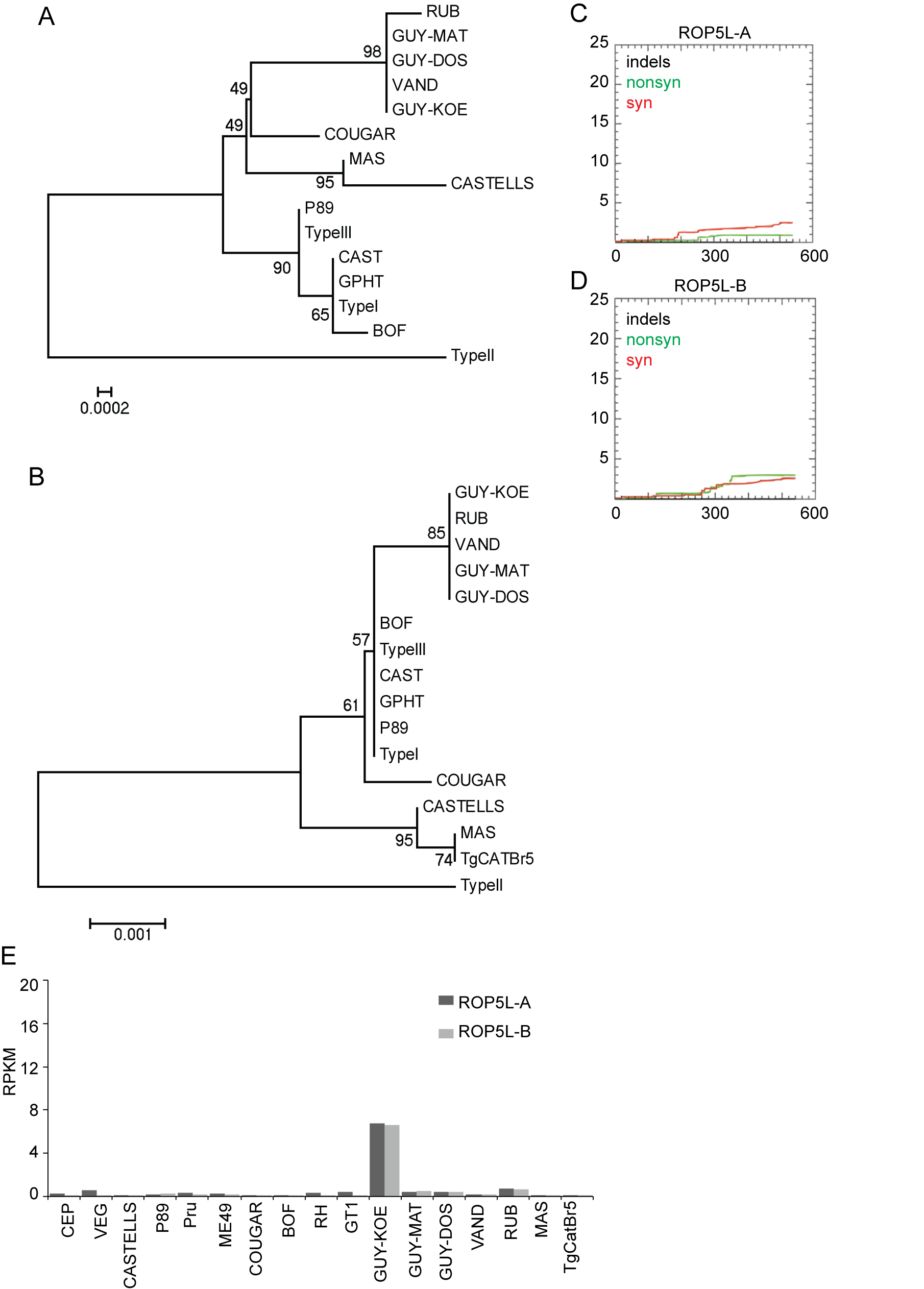

Supplement: Figure S3 — Phylogenetic analysis and expression of ROP5L-A and B. (A) ROP5L-A and B (B) phylogenetic analysis of coding nucleotide sequences by Neighbor Joining and cumulative mutations codon by codon by type (C and D respectively). E) Expression by RNA-Seq analysis of 24 hour infection with indicated strains in bone marrow-derived macrophages. (TIF) [file ppat.1002784.s003.tif]

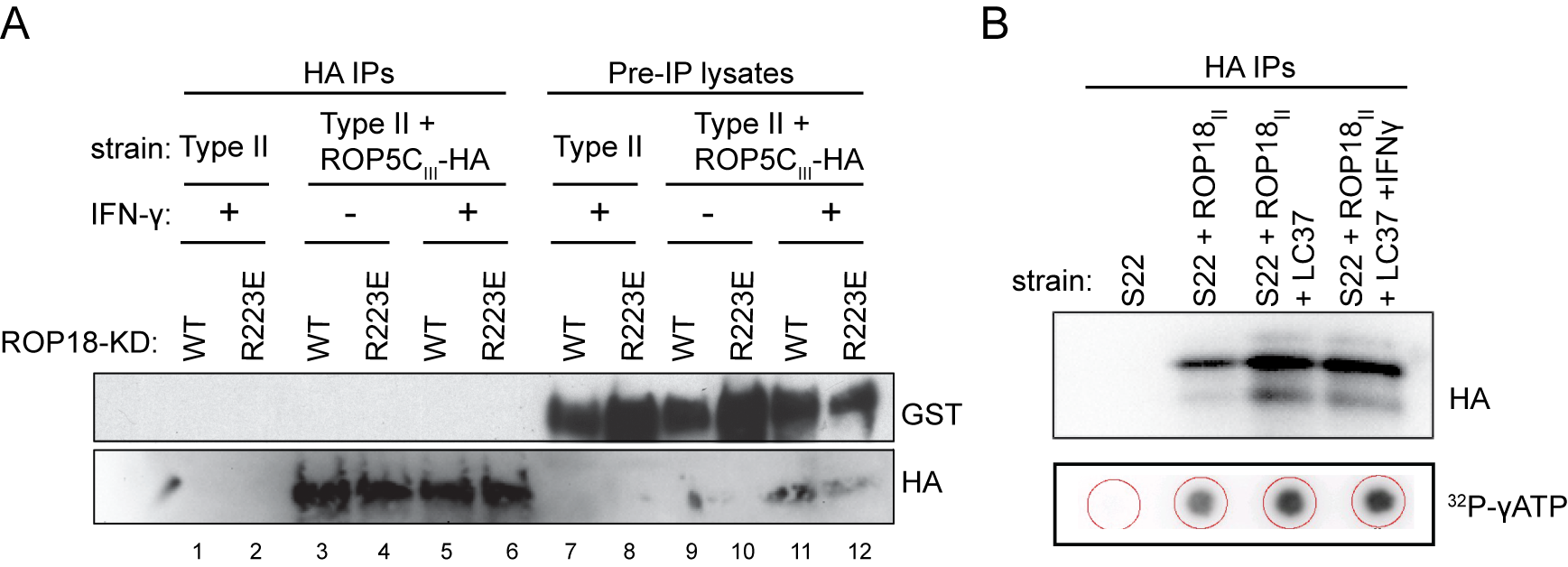

Supplement: Figure S5 — ROP5 does not directly interact with ROP18 and is not necessary for ROP18 kinase activity. (A) Wild-type (wt) or mutant R223E recombinant proteins comprising the kinase domain (KD) of ROP18I fused to MBP-GST were added to lysates prepared from IFNγ-stimulated and unstimulated MEFs infected with type II or type II + ROP5CIII-HA parasites and incubated for 30 minutes before immunoprecipitating the reactions with anti-HA. Both the immunoprecipitates (lanes 1–6) and pre-IP lysates (lanes 7–12) were Western blotted with anti-GST and anti-HA. (B) Kinase activity of ROP18-HA immunoprecipitated from IFNγ-stimulated or unstimulated MEFs infected with S22, S22 + ROP18II-HA or S22 + LC37 + ROP18II-HA parasite strains. Half of the immunoprecipitated protein was Western blotted with anti-HA (top). The remaining immunoprecipitated proteins were incubated with 32P-γ-ATP and a model peptide substrate (Lim, D., submitted) and spotted in quadruplicate onto phospho-cellulose paper where the 32P-γ-ATP incorporation was quantified by phosphorimage analysis (bottom). This experiment was performed twice and the graph represents the mean from those experiments. (TIF) [file ppat.1002784.s005.tif]

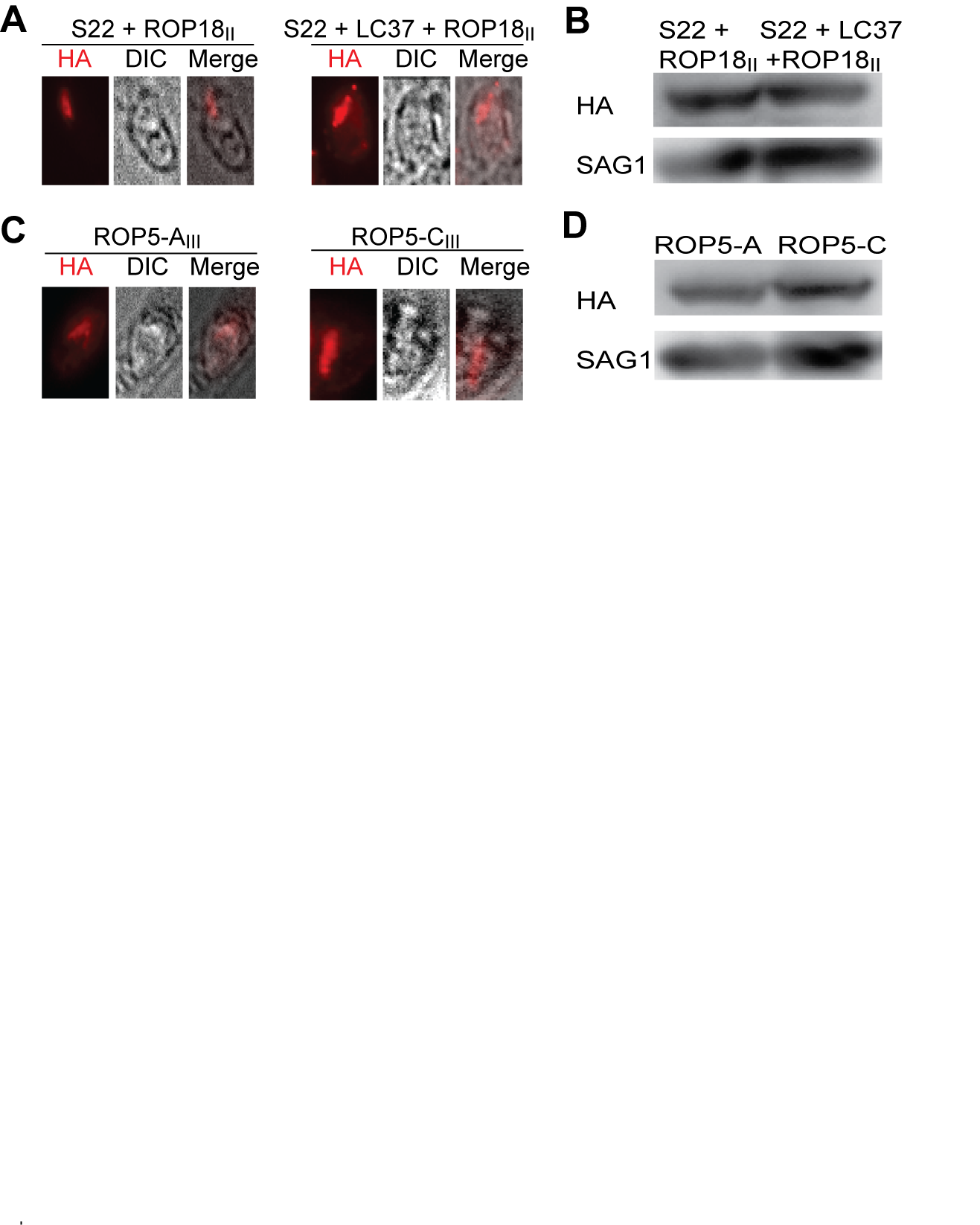

Supplement: Figure S6 — Expression and localization of transgenic ROP18 and ROP5. (A) Immunofluorescence of HA (red) in S22 + ROP18II-HA and S22 + LC37 + ROP18II-HA parasites as well as DIC and merged images. (B) Western blot for HA (top) and SAG1 (bottom) comparing expression of S22 + ROP18II-HA and S22 + LC37 + ROP18II-HA strains used for mouse infections. (C) Immunofluorescence of HA (red) in Pru + ROP5-AIII-HA and Pru + ROP5-CIII-HA parasites as well as DIC and merged images. (D) Western blot for HA (top) and SAG1 (bottom) comparing expression of Pru + ROP5-AIII-HA and Pru + ROP5-CIII-HA strains used for mouse infections. (TIF) [file ppat.1002784.s006.tif]
