# Supplementary material for: The Rhoptry Proteins ROP18 and ROP5 Mediate Toxoplasma gondii Evasion of the Murine, But Not the Human, Interferon-Gamma Response
Source: PLoS Pathog. 2012 Jun 28;8(6):e1002784. doi: 10.1371/journal.ppat.1002784 (PMC3386190; doi:10.1371/journal.ppat.1002784)
Supplement: Figure S4 — Amino acid alignments of sequenced ROP5 genes. Amino acid alignments of ROP5-A and B/C as well as ROP5L-A and B for sequenced strains. (PDF) [file ppat.1002784.s004.pdf]

ROP5-A

```
Type I    MATKLARLAT WLVLVGCLLW RAGAVQLSPP NSRTNDLASG TPHVARGDTE
Type III .......... .......... .......... .......... ..........
Type II  .......... .......... .......... .......... ..........
COUGAR   .......... .......... .......... .......... ..........
CAST     .......... .......... .......... .......... ..........
GPHT     .......... .......... .......... .......... ..........
P89      .......... .......... .......... .......... ..........
VAND     .......... .......... .......... .......... ..........
GUY-KOE  .......... .......... .......... .......... ..........
GUY-DOS  .......... .......... .......... .......... ..........
GUY-MAT  .......... .......... .......... .......... ..........
RUB      .......... .......... .......... .......... ..........
CASTELLS .......... .......... .......... .......... ..........

Type I    AQSGTGDDSD FPQAVAEEVA DMSGGRVPRV PASSTTTSAS EGIFRRLVRR
Type III .......... .......... .......... .......... ..........
Type II  .......... ...G.V.... .......... .......... ..........
COUGAR   .......... .....V.... .......... .......... ..........
CAST     .......... .......... .......... .......... ..........
GPHT     .......... .......... .......... .......... ..........
P89      .......... .......... .......... .......... ..........
VAND     .......... .....V.... .......... ........E. ..........
GUY-KOE  .......... .....V.... .......... ........E. ..........
GUY-DOS  .......... .....V.... .......... ........E. ..........
GUY-MAT  .......... .....V.... .......... .......... ..........
RUB      .......... .....V.... .......... .......... ..........
CASTELLS .......... .....V.... .......... .......... ..........

Type I    LRRGRGTADG AGVADETHQE PRPPLRKRLA QHFRRLRGFF GRLTPRWLSG
Type III .......... .......... .......... .......... ..........
Type II  .......... .........G .......... .......... ..........
COUGAR   .......... .........G .......... .......... ..........
CAST     .......... .......... .......... .......... ..........
GPHT     .......... .......... .......... .......... ..........
P89      .......... .......... .......... .......... ..........
VAND     .......... .........G .......... .......... ..........
GUY-KOE  .......... .........G .......... .......... ..........
GUY-DOS  .......... .........G .......... .......... ..........
GUY-MAT  .......... .........G .......... .......... ..........
RUB      .......... .........G .......... .......... ..........
CASTELLS .......... .........G ..T....... .......... ..........

Type I    LGRRAQRWWR GRQRPLLDPS FHGLEAGDSF MRDLLKREKE LIGYCREEAL
Type III .......... .......... .......... .......... ..........
Type II  .......... .......... .......... ......H... ..........
COUGAR   .......... .......... .......... ......H.E. ..........
CAST     .......... .......... .......... .......... ..........
GPHT     .......... .......... .......... .......... ..........
P89      .......... .......... .......... ........E. ..........
VAND     .......... .......... .......... ......H.E. ..........
GUY-KOE  .......... .......... .......... ......H.E. ..........
GUY-DOS  .......... .......... .......... ......H.E. ..........
```

```
GUY-MAT    .......... .......... .......... ......H.E. ..........
RUB        .......... .......... .......... ......H.E. ..........
CASTELLS   ..L....... .......... .......... ........E. ..........

Type I     KEPAAMVEAV TATVWPQNAE TTVDSLLSQG ERKLKLVQPL RVGDRSVVFL
Type III   .......... .......... .......... .......... ..........
Type II    .......... M......... .......... .......E.. ..........
COUGAR     .......... M....Q..D. .......... .......E.. ..........
CAST       .......... .......... .......... .......... ..........
GPHT       .......... .......... .......... .......... ..........
P89        .......... .......... .......... .......... ..........
VAND       .......... M.......D. .......... .......E.. ..........
GUY-KOE    .......... M.......D. .......... .......E.. ..........
GUY-DOS    .......... ........D. .......... .......... ..........
GUY-MAT    .......... M.......D. .......... .......E.. ..........
RUB        .......... M.......D. .......... .......E.. ..........
CASTELLS   .......... M......... .......... .......E.. ..........

Type I     VRDVERLEYF ALKVFTMGAE NSRSELERLH EATFAAARLL GESPEEARDR
Type III   .......... .......... .......... .......... ..........
Type II    .......... .......... .......... .......... ..........
COUGAR     .......... .......... .......... .......... ..........
CAST       .......... .......... .......... .......... ..........
GPHT       .......... .......... .......... .......... ..........
P89        .......... .......... .......... .......... ..........
VAND       .......... .......... .......... .......... ..........
GUY-KOE    .......... .......... .......... .......... ..........
GUY-DOS    .......... .......... .......... .......... ..........
GUY-MAT    .......... .......... .......... .......... ..........
RUB        .......... .......... .......... .......... ..........
CASTELLS   .......... .......... .......... .......... ..........

Type I     RRLLLPSDVV AVQSQPPFAQ LSPGQSDYAV ANYLLLMPAA SVDLELLFST
Type III   .......... .......... .......... .......... ..........
Type II    ........A. .......... .......... ...F...... ........R.
COUGAR     ........A. .......... .......... ...F...... ........R.
CAST       .......... .......... .......... .......... ..........
GPHT       .......... .......... .......... .......... ..........
P89        .......... .......... .......... .......... ..........
VAND       ........A. .......... .......... ...F...... .......IRP
GUY-KOE    ........A. .......... .......... ...F...... .......IRP
GUY-DOS    ........A. .......... .......... ...F...... .......IRP
GUY-MAT    ........A. .......... .......... ...F...... .......IRP
RUB        ........A. .......... .......... ...F...... .......IRP
CASTELLS   ........A. .......... .......... ...F...... .......IRS

Type I     LNFVYVFRGG EGILARHILT AQLIRLAANL QSKGLVHGRF TPDNLFLMPY
Type III   .......... .......... .......... .......... ..........
Type II    .D.......E .......L.. .......... .......... ........G.
COUGAR     .D.A..L..E .....P.... .......... .......... ..........
CAST       .......... .......... .......... .......... ..........
GPHT       .......... .......... .......... .......... ..........
P89        .......... .......... .......... .......... ..........
VAND       AD.GDI.S.Q Q.R..L.... .......... .......... ..........
```

```
GUY-KOE  AD.GDI.S.Q Q.R..L.... .......... .......... ..........
GUY-DOS  AD.GDI.S.Q Q.R..L.... .......... .......... ..........
GUY-MAT  AD.GDI.S.Q Q.R..L.... .......... .......... ..........
RUB      AD.GDI.S.Q Q.R..L.... .......... .......... ..........
CASTELLS AD..D..S.Q Q.R..TS... .......... .......... ..........

Type I   GPVMLGDVSA LWKVGTRGPA SSVPVTYAPR EFLNANTATF THALNAWQLG
Type III .......... .......... .......... .......... ..........
Type II  .......A.. .......... .......... .......... ..........
COUGAR   .......A.. .......... .......... .......... ..........
CAST     .......... .......... .......... .......... ..........
GPHT     .......... .......... .......... .......... ..........
P89      .......... .......... .......... .......... ..........
VAND     .......A.. .......... .......... .......... ..........
GUY-KOE  .......A.. .......... .......... .......... ..........
GUY-DOS  .......A.. .......... .......... .......... ..........
GUY-MAT  .......A.. .......... .......... .......... ..........
RUB      .......A.. .......... .......... .......... ..........
CASTELLS .......A.. .R........ .......... .......... ..........

Type I   LSIYRVWCLF LPFGLVTPGI KGSWKRPSLL VPGTDSLSFF PCAPVPDFVE
Type III .........V .......... .......... .......... ..........
Type II  .........V .......... .RT......R .......L.D S.I......Q
COUGAR   .........V .......... .........R .......A.. S.TRL....Q
CAST     .......... .......... .......... .......... ..........
GPHT     .........V .......... .......... .......... ..........
P89      .........V .......... .......... .......... ..........
VAND     .........V .......... .........R .......P.. S.TRL....Q
GUY-KOE  .........V .......... .........R .......P.. S.TRL....Q
GUY-DOS  .........V .......... .........R .......P.. S.TRL....Q
GUY-MAT  .........V .......... .........R .......P.. S.TRL....Q
RUB      .........V .......... .........R .......P.. S.TRL....Q
CASTELLS .IL......V .......... .........G .......A.G S.T......Q

Type I   TLIRRFLNFD RRRRLLPLEA METPEFLQLQ NEISRRLSTG QPTAAPSVA*
Type III .......... .......... .......... .......... ..........
Type II  ..T....... .......... .......... ....SS.... ..........
COUGAR   .......... .......... .......... .....S.... ..........
CAST     .......... .......... .......... .......... ..........
GPHT     .......... .......... .......... .......... ..........
P89      .......... .......... .......... .......... ..........
VAND     .......... .......... .......... .....S.... ..........
GUY-KOE  .......... .......... .......... .....S.... ..........
GUY-DOS  .......... .......... .......... .....S.... ..........
GUY-MAT  .......... .......... .......... .....S.... ..........
RUB      .......... .......... .......... .....S.... ..........
CASTELLS .......... .......... .......... ....SN.... ..........
```

ROP5-B/C

```
Type III B    MATKLARLAT WLVLVGCLLW RAGAVQLSPP NSRTNDLASG TPHVARGDTE
Type III C    .......... .......... .......... .......... ......R...
Type I B      .......... .......... .......... .......... ..........
Type I C      .......... .......... .......... .......... ..........
Type II B     .......... .......... .......... .......... ..........
Type II C     .......... .......... .......... .......... ..........
P89 B.1       .......... .......... .......... .......... ..........
CAST B.1      .......... .......... .......... .......... ..........
GPHT B.1      .......... .......... .......... .......... ..........
GPHT B.2      .......... .......... .......... .......... ..........
P89 B.2       .......... .......... .......... .......... ..........
CAST B.2      .......... .......... .......... .......... ..........
BOF B         .......... .......... .......... .......... ..........
GUYDOS B.2    .......... .......... .......... .......... ..........
GUYMAT B.2    .......... .......... .......... .......... ..........
GUYKOE B.1    .......... .......... .......... .......... ..........
GUYMAT B.1    .......... .......... .......... .......... ..........
RUB B.1       .......... .......... .......... .......... ..........
GUYKOE B.2    .......... .......... .......... .......... ..........
GUYKOE B.4    .......... .......... .......... .......... ..........
VAND B.3      .......... .......... .......... .......... ..........
RUB B.2       .......... .......... .......... .......... ..........
VAND B.2      .......... .......... .......... .......... ..........
GUYKOE B.3    .......... .......... .......... .......... ..........
GUYDOS B.1    .......... .......... .......... .......... ..........
GUYMAT B.3    .......... .......... .......... .......... ..........
COUGAR B      .......... .......... .......... .......... ..........
GUYDOS B.3    .......... .......... .......... .......... ..........
VAND B.1      .......... .......... .......... .......... ..........
CASTELLS B.1  .......... .......... .......... .......... ..........
TgCatBr5 B.2  .......... .......... .......... .......... ..........
MAS B.2       .......... .......... .......... .......... ..........
TgCatBr5 B.1  .......... .......... .......... .......... ..........
CASTELLS B.2  .......... .......... .......... .......... ..........
MAS B.1       .......... .......... .......... .......... ..........

Type III B    AQSGTGDDSD FPQAVVEEVA DMSGGRVPRV PASSTTTSAS EGIFRRLVRR
Type III C    .......... .....A.... .......... .......... ..........
Type I B      .......... .......... .......... .......... ..........
Type I C      .......... .....A.... .......... .......... ..........
Type II B     .......... ...G...... .......... .......... ..........
Type II C     .......... ...G...... .......... .......... ..........
P89 B.1       .......... .......... .......... .......... ..........
CAST B.1      .......... .......... .......... .......... ..........
GPHT B.1      .......... .......... .......... .......... ..........
GPHT B.2      .......... .....A.... .......... .......... ..........
P89 B.2       .......... .....A.... .......... .......... ..........
CAST B.2      .......... .....A.... .......... .......... ..........
BOF B         .......... .....A.... .......... .......... ..........
GUYDOS B.2    .......... .....A.... .......... .......... ..........
GUYMAT B.2    .......... .....A.... .......... .......... ..........
GUYKOE B.1    .......... .....A.... .......... .......... ..........
GUYMAT B.1    .......... .....A.... .......... .......... ..........
RUB B.1       .......... .....A.... .......... .......... ..........
GUYKOE B.2    .......... .....A.... .......... .......... ..........
```

```
GUYKOE B.4    .......... .....A.... .......... .......... ..........
VAND B.3      .......... .....A.... .......... .......... ..........
RUB B.2       .......... .......... .......... ........E. ..........
VAND B.2      .......... .......... .......... ........E. ..........
GUYKOE B.3    .......... .......... .......... ........E. ..........
GUYDOS B.1    .......... .......... .......... ........E. ..........
GUYMAT B.3    .......... .......... .......... ........E. ..........
COUGAR B      .......... .......... .......... ....A..... ..........
GUYDOS B.3    .......... .....A.... .......... .......... ..........
VAND B.1      .......... .......... .......... ........E. ..........
CASTELLS B.1 .......... .......... .......... .......... ..........
TgCatBr5 B.2 .......... .......... .......... .......... ..........
MAS B.2       .......... .......... .........M .......... ..........
TgCatBr5 B.1 .......... .......... .........M .......... ..........
CASTELLS B.2 .......... .......... .........M .......... ..........
MAS B.1       .......... .......... .......... .......... ..........

Type III B    LRRGRGTADG AGVADETHQG PRPPLRKRLA QHFRRLRGFF GRLTPRWLSG
Type III C    .......... .........E .......... .......... ..........
Type I B      .......... .......... .......... .......... ..........
Type I C      .......... .........E .......... .......... ..........
Type II B     .......... .......... .......... .......... ..........
Type II C     .......... .......... .......... .......... ..........
P89 B.1       .......... .......... .......... .......... ..........
CAST B.1      .......... .......... .......... .......... ..........
GPHT B.1      .......... .......... .......... .......... ..........
GPHT B.2      .......... .........E .......... .......... ..........
P89 B.2       .......... .........E .......... .......... ..........
CAST B.2      .......... .........E .......... .......... ..........
BOF B         .......... .........E .......... .......... ..........
GUYDOS B.2    .......... .......... .......I.. .......... ..........
GUYMAT B.2    .......... .......... .......I.. .......... ..........
GUYKOE B.1    .......... .......... .......... .......... ..........
GUYMAT B.1    .......... .......... .......I.. .......... ..........
RUB B.1       .......... .......... .......I.. .......... ..........
GUYKOE B.2    .......... .......... .......I.. .......... ..........
GUYKOE B.4    .......... .......... .......... .......... ..........
VAND B.3      .......... .......... .......I.. .......... ..........
RUB B.2       .......... .......... .......... .......... ..........
VAND B.2      .......... .......... .......... .......... ..........
GUYKOE B.3    .......... .......... .......... .......... ..........
GUYDOS B.1    .......... .......... .......... .......... ..........
GUYMAT B.3    .......... .......... .......... .......... ..........
COUGAR B      .......... .......... .......... .......... ..........
GUYDOS B.3    .......... .......... .......I.. .......... ..........
VAND B.1      .......... .......... .......... .......... ..........
CASTELLS B.1 .......... .........R ..T....... .......... ..........
TgCatBr5 B.2 .......... .........R ..T....... .......... ..........
MAS B.2       .......... .......... ..T....... .......... ..........
TgCatBr5 B.1 .......... .......... ..T....... .......... ..........
CASTELLS B.2 .......... .......... ..T....... .......... ..........
MAS B.1       .......... .......... ..T....... .......... ..........

Type III B    LGRRAQRWWR GRQRPLLDPS FHGLEAGDSF MRDLLKREEE LIGYCREEAL
Type III C    .......... .......... .......... .......... ..........
Type I B      .......... .......... .......... .......... ..........
Type I C      .......... .......... .......... .......... ..........
```

```
Type II B     .......... .......... .......... .......... ......K...
Type II C     .......... .......... .......... .......... ..........
P89 B.1       .......... .......... .......... .......... ..........
CAST B.1      .......... .......... .......... .......... ..........
GPHT B.1      .......... .......... .......... .......... ..........
GPHT B.2      .......... .......... .......... .......... ..........
P89 B.2       .......... .......... .......... .......... ..........
CAST B.2      .......... .......... .......... .......... ..........
BOF B         .......... .......... .......... .......... ..........
GUYDOS B.2    .......... .......G.. .......... .......... ..........
GUYMAT B.2    .......... .......G.. .......... .......... ..........
GUYKOE B.1    .......... .......... .......... .......... ..........
GUYMAT B.1    .......... .......G.. .......... .......... ..........
RUB B.1       .......... .......... .......... .......... ..........
GUYKOE B.2    .......... .......G.. .......... .......... ..........
GUYKOE B.4    .......... .......... .......... .......... ..........
VAND B.3      .......... .......G.. .......... .......... ..........
RUB B.2       .......... .......... .......... .....E...A ..........
VAND B.2      .......... .......... .......... .....E...A ..........
GUYKOE B.3    .......... .......... .......... .....E...A ..........
GUYDOS B.1    .......... .......... .......... .....E...A ..........
GUYMAT B.3    .......... .......... .......... .....E...A ..........
COUGAR B      .......... .......... .......... .........A ..........
GUYDOS B.3    .......... .......G.. .......... .......... ..........
VAND B.1      .......... .......... .......... ......HD-- ..........
CASTELLS B.1  .......... .......... .......... .....T.... ..........
TgCatBr5 B.2  .......... .......... .......... .....T.... ..........
MAS B.2       .......... .......... .......... .....T.... ..........
TgCatBr5 B.1  .......... .......... .......... .....T.... ..........
CASTELLS B.2  ..L....... .......... .......... .....T.... ..........
MAS B.1       .......... .......... .......... .......... ..........

Type III B    KEPAAMVEAV TATVWPQNAE TTVDSLLSQG ERKLKLVEPL RVGDRSVVFL
Type III C    .......... .......... .......... .......... ..........
Type I B      .......... .......... .......... .......... ..........
Type I C      .......... .......... .......... .......... ..........
Type II B     E......... .....Q..D. .......... .......Q.. ..........
Type II C     .......... M......... .......... .......... ..........
P89 B.1       .......... .......... .......... .......... ..........
CAST B.1      .......... .......... .......... .......... ..........
GPHT B.1      .......... .......... .......... .......... ..........
GPHT B.2      .......... .......... .......... .......... ..........
P89 B.2       .......... .......... .......... .......... ..........
CAST B.2      .......... .......... .......... .......... ..........
BOF B         .......... .......... .......... .......... ..........
GUYDOS B.2    .......... .......... .......... .......... ..........
GUYMAT B.2    .......... .......... .......... .......... ..........
GUYKOE B.1    .......... .......... .......... .......... ..........
GUYMAT B.1    .......... .......... .......... .......Q.. ..........
RUB B.1       .......... .......... .......... .......Q.. ..........
GUYKOE B.2    .......... .......... .......... .......Q.. ..........
GUYKOE B.4    .......... .......... .......... .......Q.. ..........
VAND B.3      .......... .......... .......... .......Q.. ..........
RUB B.2       .......A.. .....Q..D. .......... .......... ..........
VAND B.2      .......A.. .....Q..D. .......... .......... ..........
GUYKOE B.3    .......A.. .....Q..D. .......... .......... ..........
GUYDOS B.1    .......A.. .....Q..D. .......... .......... ..........
```

```
GUYMAT B.3    .......A.. .....Q..D. .......... .......... ..........
COUGAR B      .......A.. .....Q..D. .......... .......... ..........
GUYDOS B.3    .......... .......... .......... .......... ..........
VAND B.1      E......A.. .......... .......... .......... ..........
CASTELLS B.1  .......... M......... .......... .......... ..........
TgCatBr5 B.2  .......... M......... .......... .......... ..........
MAS B.2       .......... M......... .......... .......... ..........
TgCatBr5 B.1  .......... M......... .......... .......... ..........
CASTELLS B.2  .......... M......... .......... .......... ..........
MAS B.1       .......... M......... .......... .......... ..........

Type III B    VRDVERLEDF ALKVFTMGAE NSRSELERLH EATFAAARLL GESPEEARDR
Type III C    .......... .......... .......... .......... ..........
Type I B      .......... .......... .......... .......... ..........
Type I C      .......... .......... .......... .......... ..........
Type II B     .......... .......... .......... .......... ..........
Type II C     .......... .......... .......... .......... ..........
P89 B.1       .......... .......... .......... .......... ..........
CAST B.1      .......... .......... .......... .......... ..........
GPHT B.1      .......... .......... .......... .......... ..........
GPHT B.2      .......... .......... .......... .......... ..........
P89 B.2       .......... .......... .......... .......... ..........
CAST B.2      .......... .......... .......... .......... ..........
BOF B         .......... .......... .......... .......... ..........
GUYDOS B.2    .......... .......... .......... .......... ..........
GUYMAT B.2    .......... .......... .......... .......... ..........
GUYKOE B.1    .......... .......... .......... .......... ..........
GUYMAT B.1    .......... .......... .......... .......... ..........
RUB B.1       .......... .......... .......... .......... ..........
GUYKOE B.2    .......... .......... .......... .......... ..........
GUYKOE B.4    .......... .......... .......... .......... ..........
VAND B.3      .......... .......... .......... .......... ..........
RUB B.2       .......... .......... .......... .....T.... ..........
VAND B.2      .......... .......... .......... .......... ..........
GUYKOE B.3    .......... .......... .......... .......... ..........
GUYDOS B.1    .......... .......... .......... .......... ..........
GUYMAT B.3    .......... .......... .......... .......... ..........
COUGAR B      .......... .......... .......... .......... ..........
GUYDOS B.3    .......... .......... .......... .......... ..........
VAND B.1      .......... .......... .......... .......... ..........
CASTELLS B.1  .......... .......... .......... .......... ..........
TgCatBr5 B.2  ........Y. .......... .......... .......... ..........
MAS B.2       .......... .......... .......... .......... ..........
TgCatBr5 B.1  .......... .......... .......... .......... ..........
CASTELLS B.2  ........Y. .......... .......... .......... ..........
MAS B.1       ........Y. .......... .......... .......... ..........

Type III B    RRLLLPSDAV AVQSQPPFAQ LSPGQDDYAV A-LFASHARC VGGS*IAL*H
Type III C    .......... .......... .....S.... .-.L...... .........D
Type I B      .......... .......... .....S.... .-.L...... .........D
Type I C      .......... .......... .....S.... .-.L...... .........D
Type II B     .......... .......... .....S.... .R..P..... .........D
Type II C     .......... .......... .....S.... .-..L..... .........D
P89 B.1       .......... .......... .......... .-........ ..........
CAST B.1      .......... .......... .......... .-........ ..........
GPHT B.1      .......... .......... .......... .-........ ..........
GPHT B.2      .......... .......... .....S.... .-.L...... .........D
```

```
P89 B.2       .......... .......... .....S.... .-.L...... .........D
CAST B.2      .......... .......... .....S.... .-.L...... .........D
BOF B         .......... .......... .....S.... .-.L...... .........D
GUYDOS B.2    .......... .......... .....S.... .-........ .........D
GUYMAT B.2    .......... .......... .....S.... .-........ .........D
GUYKOE B.1    .......... .......... .....S.... .-........ .........G
GUYMAT B.1    .......... .......... .....S.N.. .-........ .......H.V
RUB B.1       .......... .......... .....S.N.. .-........ .......H.V
GUYKOE B.2    .......... .......... .....S.N.. .-........ .......H.V
GUYKOE B.4    .......... .......... .....S.N.. .-........ .......H.V
VAND B.3      .......... .......... .....S.N.. .-........ .......H.V
RUB B.2       .......... .......... .....S.... .-..P..... .........D
VAND B.2      .......... .......... .....S.... .-..P..... .........D
GUYKOE B.3    .......... .......... .....S.... .-..P..... .........D
GUYDOS B.1    .......... .......... .....S.... .-..P..... .........D
GUYMAT B.3    .......... .......... .....S.... .-..P..... .........D
COUGAR B      .......... .......... .....S.... .-..P..... .......P.D
GUYDOS B.3    .......... .......... .....S.... .-........ .........D
VAND B.1      .......... .......... .....S.... .-..P..... ......V..E
CASTELLS B.1  .......... .......... .....S.... .-..P..... .........V
TgCatBr5 B.2  .......... .......... .....S.... .-..P..... .........V
MAS B.2       .......... .......... .....S.... .-........ ...FG..H.V
TgCatBr5 B.1  .......... .......... .....S.... .-........ ...FG..H.V
CASTELLS B.2  .......... .......... .....S.... .-........ ...FG..H.V
MAS B.1       .......... .......... .....S.... .-..P..... .......H.V

Type III B    IGLRVCIQGG RRYFSASHTN GTADPSGSQP AEQRTCAWTF HTG*PFHYAR
Type III C    ..FP..T.EY ..F....... .......... .......... ..........
Type I B      ..FP..T.EY ..F....... .......... .......... ..........
Type I C      ..FP..T.EY ..F....... .......... .......... ..........
Type II B     .......... .......L.. .......... .........L ..*K......
Type II C     .......... .......L.. .......... .........L ..*K......
P89 B.1       .......... .......... .......... .......... ..........
CAST B.1      .......... .......... .......... .......... ..........
GPHT B.1      .......... .......... .......... .......... ..........
GPHT B.2      ..FP..T.EY ..F....... .......... .......... ..........
P89 B.2       ..FP..T.EY ..F....... .......... .......... ..........
CAST B.2      ..FP..T.EY ..F....... .......... .......... ..........
BOF B         ..FP..T.EY ..F....... .......... .......... ..........
GUYDOS B.2    ..F......Y ..S....... .......... .......... ..........
GUYMAT B.2    ..F......Y ..S....... .R........ .......... ..........
GUYKOE B.1    ..HS.....Y ..S....... .......... .......... ..........
GUYMAT B.1    R...GY.RRA A.MVNDF..Y .......... .......... ..........
RUB B.1       R...GY.RRA A.MVNDF..Y .......... .......... ..........
GUYKOE B.2    R...GY.RRA A.MVNDF..Y .......... .......... ..........
GUYKOE B.4    R...GY.RRA A.MVNDF..Y .......... .......... ..........
VAND B.3      R...GY.RRA A.MVNDF..Y .......... .......... ..........
RUB B.2       ......T... .......... .......... .......... ..........
VAND B.2      ......T... .......... .......... .......... ..........
GUYKOE B.3    ......T... .......... .......... .......... ..........
GUYDOS B.1    ......T... .......... .......... .......... ..........
GUYMAT B.3    ......T... .......... .......... .......... ..........
COUGAR B      ......T... .......... .......... .......... ..........
GUYDOS B.3    ..F...T... .......... .......... .......... ..........
VAND B.1      ..P..R.... .......... .......... .......... ..*.......
CASTELLS B.1  ......T.E* ......P..S .......... .........L ..........
TgCatBr5 B.2  ......T.E* ......P..S .......... .........L ..........
```

```
MAS B.2       S...GY..RA A.T..DF... .......... .........L ..........
TgCatBr5 B.1 S...GY..RA A.T..DF... .......... .........L ..........
CASTELLS B.2 S...GY..RA A.T..DF... .......... .........L ..........
MAS B.1       S...GY..RA A.T..DF... .......... .........L ..........

Type III B    WPPDAGGCIR IVEGRNPRTG IKRPGYLCAS RVLECKHGNI YTRAQCVATG
Type III C    .......... VE........ .......... .......... ....G.....
Type I B      .......... VE........ .......... .......... ....G.....
Type I C      .......... VE........ .......... .......... ....G.....
Type II B     ....D....H VE........ .......... G......... ..........
Type II C     ....D....H VE........ .......... G......... ..........
P89 B.1       .......... .......... .......... .......... ..........
CAST B.1      .......... .......... .......... .......... ..........
GPHT B.1      .......... .......... .......... G......... ....G.....
GPHT B.2      .......... VE........ .......... .......... ....G.....
P89 B.2       .......... VE........ .......... .......... ....G.....
CAST B.2      .......... VE........ .......... .......... ....G.....
BOF B         .......... VE........ .......... .......... ....G.....
GUYDOS B.2    .......... VE........ .......... .......... ....G.....
GUYMAT B.2    .......... VE........ .......... .......... ....G.....
GUYKOE B.1    .......... VE........ .......... .......... ....G.....
GUYMAT B.1    .......... VE........ .......... .......... ....R.....
RUB B.1       .......... VE........ .......... .......... ....R.....
GUYKOE B.2    .......... VE........ .......... .......... ....G.....
GUYKOE B.4    .......... VE........ .......... .......... ....G.....
VAND B.3      .......... VE........ .......... .......... ....G.....
RUB B.2       .......... VE........ .......... G......... ..........
VAND B.2      .......... VE........ .......... G......... ..........
GUYKOE B.3    .......... VE........ .......... G......... ..........
GUYDOS B.1    .......... VE........ .......... G......... ..........
GUYMAT B.3    .......... VE........ .......... G......... ..........
COUGAR B      .......... VE........ .......... G......... ..........
GUYDOS B.3    .......... VE........ .......... G......... ........?.
VAND B.1      .......... VE........ .......... G......... ..........
CASTELLS B.1 .......... VE........ .......... G......... ..........
TgCatBr5 B.2 .......... VE........ .......... G......... ..........
MAS B.2       .......... VE........ .......... G......... ..........
TgCatBr5 B.1 .......... VE........ .......... G......... ..........
CASTELLS B.2 .......... VE........ .......... G......... ..........
MAS B.1       .......... VE........ .......... G......... ..........

Type III B    S*HIPGLVPI LAFRTRDTWD QRVMEKAKST SSRD*QSGIR LMYT-SA*LR
Type III C    .......... .......... ......T... .......... ....-.....
Type I B      .......... .......... ......T... .......... ....-.....
Type I C      .......... .......... ......T... .......... ....-.....
Type II B     .........S .......... .TD....... .......A.. ....-C....
Type II C     .........S .......... .TDT...... .......A.. ....-C....
P89 B.1       .......... .......... .......... .......... ....-.....
CAST B.1      .......... .......... .......... .......... ....-.....
GPHT B.1      .......... .......... .......... .......... ....-.....
GPHT B.2      .......... .......... ......T... .......... ....-.....
P89 B.2       .......... .......... ......T... .......... ....-.....
CAST B.2      .......... .......... ......T... .......... ....-.....
BOF B         .......... .......... ......T... .......... ....-.....
GUYDOS B.2    .........S .......... .T........ ......C..L ..*RRC....
GUYMAT B.2    .........S .......... .T........ ......C..L ..*RRC....
GUYKOE B.1    .........S .......... .T........ ......C..L ..*RRC....
```

```
GUYMAT B.1      .........S .......... .T........ .......V.L P.WI-C....
RUB B.1         .........S .......... .T........ .......V.L P.WI-C....
GUYKOE B.2      .........S .......... .T........ .......V.L P.WI-C....
GUYKOE B.4      .........S .......... .T........ .......V.L P.WI-C....
VAND B.3        .........S .......... .T........ .......V.L P.WI-C....
RUB B.2         ...T.....S .......... .......... .......L.. ..W.-.....
VAND B.2        ...T.....S .......... .......... .......L.. ..W.-.....
GUYKOE B.3      ...T.....S .......... .......... .......L.. ..W.-.....
GUYDOS B.1      ...T.....S .......... .......... .......L.. ..W.-.....
GUYMAT B.3      ...T.....S .......... .......... .......L.. ..W.-.....
COUGAR B        ...T.....S .......... .......... .......L.. ..W.-.....
GUYDOS B.3      ...T.....S .......... .......... .......L.. ..W.-.....
VAND B.1        .........S .......... .......... .......A.. ....-C....
CASTELLS B.1    .YL....... .......... .........R .......... ....-.....
TgCatBr5 B.2    .YL....... .......... .........R .......... ....-.....
MAS B.2         .YL.....S. .......... .........R .......... ....-.....
TgCatBr5 B.1    .YL.....S. .......... .........R .......... ....-.....
CASTELLS B.2    .YL....... .......... .........R .......... ....-.....
MAS B.1         .YL....... .......... .........R .......... ....-.....

Type III B      EDTYWTVPQL R*ASTPASLG GHGDARVPPA PKRNIEQPIN RTTYCCALSR
Type III C      A......... .......... .......... .......... ...H......
Type I B        A......... .......... .......... .......... ...H......
Type I C        A......... .......... .......... .......... ...H......
Type II B       A...*..... ...A...... .......... .......... ..........
Type II C       A...*..... ...A...... .......... .......... ..........
P89 B.1         .......... .......... .......... .......... ..........
CAST B.1        .......... .......... .......... .......... ..........
GPHT B.1        .......... .......... .......... .......... ..........
GPHT B.2        A......... .......... .......... .......... ...H......
P89 B.2         A......... .......... .......... .......... ...H......
CAST B.2        A......... .......... .......... .......... ...H......
BOF B           A......... .......... .......... ......A... ..........
GUYDOS B.2      A...*..... ...A.H.... .......... ..Q...E... S..H......
GUYMAT B.2      A...*..... ...A.H.... .......... ..Q...E... ...H......
GUYKOE B.1      A...*..... ...A.H.... .......... ..Q...E... S..H......
GUYMAT B.1      .......... ...A...... .......... ..Q....... ..IH......
RUB B.1         .......... ...A...... .......... ..Q....... ..IH......
GUYKOE B.2      .......... ...A...... .......... .......... ..IH......
GUYKOE B.4      .......... ...A...... .......... .......... ..IH......
VAND B.3        .......... ...A...... .......... ......E... ..........
RUB B.2         A......... ...A...P.. .......... ......E... ..........
VAND B.2        A......... ...A...P.. .......... ......E... ..........
GUYKOE B.3      A......... ...A...P.. .......... ......E... ..........
GUYDOS B.1      A......... ...A...P.. .......... ......E... ..........
GUYMAT B.3      A......... ...A...P.. .......... ......E... ..........
COUGAR B        A......... ...A...P.. .......... ......E... ..........
GUYDOS B.3      A......... ...A...P.. .......... ......E... ..........
VAND B.1        A..D*..... ...A...P.. .......... ..Q...A..I .SN.......
CASTELLS B.1    G...*..... ...A...... .......... .......... ..........
TgCatBr5 B.2    G...*..... ..GA...... .......... .......... ....R.....
MAS B.2         A......... ...A...... .......... .......... ..........
TgCatBr5 B.1    A......... ...A...... .......... .......... ..........
CASTELLS B.2    A......... ...A...... .......... .......... ..........
MAS B.1         A......... ...A...... .......... .......... ..........

Type III B      L
```

```
Type III C    .
Type I B      .
Type I C      .
Type II B     .
Type II C     .
P89 B.1       .
CAST B.1      .
GPHT B.1      .
GPHT B.2      .
P89 B.2       .
CAST B.2      .
BOF B         .
GUYDOS B.2    .
GUYMAT B.2    .
GUYKOE B.1    .
GUYMAT B.1    .
RUB B.1       .
GUYKOE B.2    .
GUYKOE B.4    .
VAND B.3      .
RUB B.2       .
VAND B.2      .
GUYKOE B.3    .
GUYDOS B.1    .
GUYMAT B.3    .
COUGAR B      .
GUYDOS B.3    .
VAND B.1      .
CASTELLS B.1 .
TgCatBr5 B.2 .
MAS B.2       .
TgCatBr5 B.1 .
CASTELLS B.2 .
MAS B.1       .
```

ROP5L-A

```
Type I    MATDARRLAT GLVLLTCLVW RAGAFQPSPP TSRANELTSG IPGEAPENTA
Type III  .......... .......... .......... .......... ..........
Type II   .......... .......... .......... .A........ ..........
MAS       .......... .......... .......... .......... ..........
TgCATBr5  .......... .......... .......... .......... ..........
CASTELLS  .......... .......... .......... .......... ..........
P89       .......... .......... .......... .......... ..........
CAST      .......... .......... .......... .......... ..........
GPHT      .......... .......... .......... .......... ..........
GUY-DOS   .......... .......... .......... .......... ..........
GUY-KOE   .......... .......... .......... .......... ..........
VAND      .......... .......... .......... .......... ..........
RUB       .......... .......... .......... .......... ..........
GUY-MAT   .......... .......... .......... .......... ..........
BOF       .......... .......... .......... .......... ..........
COUGAR    .......... .......... .......... .......... ..........

Type I    EERGIGDISD YPHSVVDDAA DISGGTVPEG PGSPASTSGS GGVFNRLFSR
Type III  .......... .......... .......... .......... ..........
Type II   .......... .......... .......... .......... ..........
MAS       .......... .......... .......... .......... ..........
TgCATBr5  .......... .......... .......... .......... ..........
CASTELLS  .......... .......... .......... .......... ..........
P89       .......... .......... .......... .......... ..........
CAST      .......... .......... .......... .......... ..........
GPHT      .......... .......... .......... .......... ..........
GUY-DOS   .......... .......... .......... .......... ..........
GUY-KOE   .......... .......... .......... .......... ..........
VAND      .......... .......... .......... .......... ..........
RUB       .......... .......... .......... .......... ..........
GUY-MAT   .......... .......... .......... .......... ..........
BOF       .......... .......... .......... .......... ..........
COUGAR    .......... .......... .......... .......... ..........

Type I    FRQVSGSTQG TGVADEPQQG PRPSLRERLA QHFRRLRVLF GRLMPRWGFG
Type III  .......... .......... .......... .......... ..........
Type II   .......... R......... .......... .......... ..........
MAS       .......... .......... .......... .......... ..........
TgCATBr5  .......... .......... .......... .......... ..........
CASTELLS  .......... .......... .......... .......... ..........
P89       .......... .......... .......... .......... ..........
CAST      .......... .......... .......... .......... ..........
GPHT      .......... .......... .......... .......... ..........
GUY-DOS   .......... .......... .......... .......... ..........
GUY-KOE   .......... .......... .......... .......... ..........
VAND      .......... .......... .......... .......... ..........
RUB       .......... .......... .......... .......... ..........
GUY-MAT   .......... .......... .......... .......... ..........
BOF       .......... .......... .......... .......... ..........
COUGAR    .......... .......... .......... .......... ..........
```

```
Type I    FGRHVGRWWP RRWPDPLFPC MEPGDEFIRS RLSQTEYRTR FYRRGMVEGY
Type III .......... .......... .......... .......... ..........
Type II  .......... .......... .......... .......... ..........
MAS      .......... .......... .......... .......... ..........
TgCATBr5 .......... .......... .......... .......... ..........
CASTELLS .......... .......... .......... .......... ..........
P89      .......... .......... .......... .......... ..........
CAST     .......... .......... .......... .......... ..........
GPHT     .......... .......... .......... .......... ..........
GUY-DOS  .......... .......... .......... .......... ..........
GUY-KOE  .......... .......... .......... .......... ..........
VAND     .......... .......... .......... .......... ..........
RUB      .......... .......... .......... .......... ..........
GUY-MAT  .......... .......... .......... .......... ..........
BOF      .......... .......... .......... .......... ..........
COUGAR   .......... .......... .......... .......... ..........

Type I   EVEVKAVTAA IWPQNTAREV ASLLDRRKRT LRVVGAFGRS VRSVLYLAQD
Type III .......... .......... .......... .......... ..........
Type II  .......... .......... .......... .......... ..........
MAS      .......... .......... .......... .......... ..........
TgCATBr5 .......... .......... .......... .......... ..........
CASTELLS .......... .......... .......... .......... ..........
P89      .......... .......... .......... .......... ..........
CAST     .......... .......... .......... .......... ..........
GPHT     .......... .......... .......... .......... ..........
GUY-DOS  .......... .......... .......... .......... ..........
GUY-KOE  .......... .......... .......... .......... ..........
VAND     .......... .......... .......... .......... ..........
RUB      .......... .......... .......... .......... ..........
GUY-MAT  .......... .......... .......... .......... ..........
BOF      .......... .......... .......... .......... ..........
COUGAR   .......... .......... .......... .......... ..........

Type I   VESQERMAIE VFTLTSGNTV SDLARVHDNL FAMTGLVSES PQQARDICRL
Type III .......... .......... .......... .......... ..........
Type II  ..T....... .......... .......... ......L... ..........
MAS      ..T....... .......... .......... .......... ..........
TgCATBr5 ..T....... .......... .......... .......... ..........
CASTELLS ..T....... .......... .......... .......... ..........
P89      .......... .......... .......... .......... ..........
CAST     .......... .......... .......... .......... ..........
GPHT     .......... .......... .......... .......... ..........
GUY-DOS  .......... .......... .......... .......... ..........
GUY-KOE  .......... .......... .......... .......... ..........
VAND     .......... .......... .......... .......... ..........
RUB      .......... .......... .......... .......... ..........
GUY-MAT  .......... .......... .......... .......... ..........
BOF      .......... .......... .......... .......... ..........
COUGAR   .......... .......... .......... .......... ..........

Type I   LLPTDAVTVP TQPPFEELNP GQSNYPVANY FLLMPPPIMS LEPLHRIVDH
Type III .......... .......... .......... .......... ..........
Type II  .......... S......... .......... .......... ..........
```

```
MAS       .......... .......... .......... .......... ..........
TgCATBr5  .......... .......... .......... .......... ..........
CASTELLS  .......... .......... .......... .......... ..........
P89       .......... .......... .......... .......... ..........
CAST      .......... .......... .......... .......... ..........
GPHT      .......... .......... .......... .......... ..........
GUY-DOS   .......... .......... .......... .......... ..........
GUY-KOE   .......... .......... .......... .......... ..........
VAND      .......... .......... .......... .......... ..........
RUB       .......... .......... .......... .......... ..........
GUY-MAT   .......... .......... .......... .......... ..........
BOF       .......... .......... .......... .......... ..........
COUGAR    .......... .......... .......... .......... ..........

Type I    EEFLTGDIGR VVRMVLTAEL IRIAANIQIR GLVHGRITSE NLFIMPDGRL
Type III  .......... .......... .......... .......... ..........
Type II   .......... .......... .......... .......... ..........
MAS       .......... .......... .......... .......... ..........
TgCATBr5  .......... .......... .......... .......... ..........
CASTELLS  .......... .......... .......... .......... ..........
P89       .......... .......... .......... .......... ..........
CAST      .......... .......... .......... .......... ..........
GPHT      .......... .......... .......... .......... ..........
GUY-DOS   .......... .......... .......... .......... ..........
GUY-KOE   .......... .......... .......... .......... ..........
VAND      .......... .......... .......... .......... ..........
RUB       .......... .......... .......... .......... ..........
GUY-MAT   .......... .......... .......... .......... ..........
BOF       .......... .......... .......... .......... ..........
COUGAR    .......... .......... .......... .......... ..........

Type I    MLGDVSALRK VGTRGPVSSV PVTYAPREFL SNTETAAFTH ALDAWQLGLV
Type III  .......... .......... .......... .......... ..........
Type II   .......... .......... .......... .......... ..........
MAS       .......... .......... .......... .......... ..........
TgCATBr5  .......... .......... .......... .......... ..........
CASTELLS  .......... .......... .......... .......... ..........
P89       .......... .......... .......... .......... ..........
CAST      .......... .......... .......... .......... ..........
GPHT      .......... .......... .......... .......... ..........
GUY-DOS   .......... .......... .......... .......... ..........
GUY-KOE   .......... .......... .......... .......... ..........
VAND      .......... .......... .......... .......... ..........
RUB       .......... .......... .......... .......... ..........
GUY-MAT   .......... .......... .......... .......... ..........
BOF       .......... .......... .......... .......... ..........
COUGAR    .......... .......... .......... .......... ..........

Type I    IHRIWCLILP FGLVTPRMKR SGRRPSMRVP GLDVLSVEAC TPMPDAVEML
Type III  .......... .......... .......... .......... ..........
Type II   .......... .......... .......... .......... ..........
MAS       .......... .......... .......... .......... ..........
TgCATBr5  .......... .......... .......... .......... ..........
CASTELLS  .......... .......... .......... .......... ..........
```

```
P89       .......... .......... .......... .......... ..........
CAST      .......... .......... .......... .......... ..........
GPHT      .......... .......... .......... .......... ..........
GUY-DOS   .......... .......... .......... .......... ..........
GUY-KOE   .......... .......... .......... .......... ..........
VAND      .......... .......... .......... .......... ..........
RUB       .......... .......... .......... .......... ..........
GUY-MAT   .......... .......... .......... .......... ..........
BOF       .......... .......... .......... .......... ..........
COUGAR    .......... .......... .......... .......... ..........

Type I    VRHFLNFNTR KRLLPLTAMG TPEFRQLQLD ISTSLSSK*
Type III  .......... .......... .......... .........
Type II   .......... .......... .......... .........
MAS       .......... .......... .......... .........
TgCATBr5  .......... .......... .......... .........
CASTELLS  .......... .......... .......... .........
P89       .......... .......... .......... .........
CAST      .......... .......... .......... .........
GPHT      .......... .......... .......... .........
GUY-DOS   .......... .......... .......... .........
GUY-KOE   .......... .......... .......... .........
VAND      .......... .......... .......... .........
RUB       .......... .......... .......... .........
GUY-MAT   .......... .......... .......... .........
BOF       .......... .......... .......... .........
COUGAR    .......... .......... .......... .........
```

ROP5L-B

```
Type I    MATDARRLAT GLVLLTCLVW RAGAFQPSPP TSRANELTSG IPGEAPENTA
Type III  .......... .......... .......... .......... ..........
Type II   .......... .......... .......... .A........ ..........
GUY-DOS   .......... .......... .......... .......... ..........
GUY-KOE   .......... .......... .......... .......... ..........
VAND      .......... .......... .......... .......... ..........
RUB       .......... .......... .......... .......... ..........
GUY-MAT   .......... .......... .......... .......... ..........
MAS       .......... .......... .......... .......... ..........
CASTELLS  .......... .......... .......... .......... ..........
COUGAR    .......... .......... .......... .......... ..........
GPHT      .......... .......... .......... .......... ..........
CAST      .......... .......... .......... .......... ..........
BOF       .......... .......... .......... .......... ..........
P89       .......... .......... .......... .......... ..........

Type I    EERGIGDISD YPHSVVDDAA DISGGTVPEG PGSPASTSGS GGVFNRLFSR
Type III  .......... .......... .......... .......... ..........
Type II   .......... .......... .......... .......... ..........
GUY-DOS   .......... .......... .......... .......... ..........
GUY-KOE   .......... .......... .......... .......... ..........
VAND      .......... .......... .......... .......... ..........
RUB       .......... .......... .......... .......... ..........
GUY-MAT   .......... .......... .......... .......... ..........
```

```
MAS       .......... .......... .......... .......... ..........
CASTELLS  .......... .......... .......... .......... ..........
COUGAR    .......... .......... .......... .......... ..........
GPHT      .......... .......... .......... .......... ..........
CAST      .......... .......... .......... .......... ..........
BOF       .......... .......... .......... .......... ..........
P89       .......... .......... .......... .......... ..........

Type I    FRQVSGSTQG TGVADEPQQG PRPLLRERLA QHFRRLRVLF GRLMPRWGFG
Type III  .......... .......... ...S...... .......... ..........
Type II   .......... R......... ...S...... .......... ..........
GUY-DOS   .......... .......... ...S...... .......... ..........
GUY-KOE   .......... .......... ...S...... .......... ..........
VAND      .......... .......... ...S...... .......... ..........
RUB       .......... .......... ...S...... .......... ..........
GUY-MAT   .......... .......... ...S...... .......... ..........
MAS       .......... .......... ...S...... .......... ..........
CASTELLS  .......... .......... ...S...... .......... ..........
COUGAR    .......... .......... ...S...... .......... ..........
GPHT      .......... .......... .......... .......... ..........
CAST      .......... .......... .......... .......... ..........
BOF       .......... .......... .......... .......... ..........
P89       .......... .......... ...S...... .......... ..........

Type I    FGRHVGRWWP RRWPDPLFPC MEPGDEFIRY LVRAKKDRIG FYRRGMVEGY
Type III  .......... .......... .......... .......... ..........
Type II   .......... .......... .......... .......... ..........
GUY-DOS   .......... .......... .......... .......... ..........
GUY-KOE   .......... .......... .......... .......... ..........
VAND      .......... .......... .......... .......... ..........
RUB       .......... .......... .......... .......... ..........
GUY-MAT   .......... .......... .......... .......... ..........
MAS       .......... .......... .......... .......... ..........
CASTELLS  .......... .......... .......... .......... ..........
COUGAR    .......... .......... .......... .......... ..........
GPHT      .......... .......... .......... .......... ..........
CAST      .......... .......... .......... .......... ..........
BOF       .......... .......... .......... .......... ..........
P89       .......... .......... .......... .......... ..........

Type I    EVEVKAVTAA IWPQNTAREV ASLLDRRKRT LRVVGAFGRS VRSVLYLAQD
Type III  .......... .......... .......... .......... ..........
Type II   .......... .......... .......... .......... ..........
GUY-DOS   .......... .......... .......... .......... ..........
GUY-KOE   .......... .......... .......... .......... ..........
VAND      .......... .......... .......... .......... ..........
RUB       .......... .......... .......... .......... ..........
GUY-MAT   .......... .......... .......... .......... ..........
MAS       .......... .......... .......... .......... ..........
CASTELLS  .......... .......... .......... .......... ..........
COUGAR    .......... .......... .......... .......... ..........
GPHT      .......... .......... .......... .......... ..........
CAST      .......... .......... .......... .......... ..........
BOF       .......... .......... .......... .......... ..........
```

```
P89       .......... .......... .......... .......... ..........

Type I    VETQEHIAAE VFTLTGESRG LDLQRVHDNL FVSAAFVAES PVLSRDRRRL
Type III  .......... .......... .......... .......... ..........
Type II   .......... .......... .......... .L....L... ..........
GUY-DOS   .......... .......... .......... ......L... ..........
GUY-KOE   .......... .......... .......... ......L... ..........
VAND      .......... .......... .......... ......L... ..........
RUB       .......... .......... .......... ......L... ..........
GUY-MAT   .......... .......... .......... ......L... ..........
MAS       .......... .......... .......... ......L... ..........
CASTELLS  .......... .......... .......... ......L... ..........
COUGAR    .......G.. .......... .......... ......L... ..........
GPHT      .......... .......... .......... .......... ..........
CAST      .......... .......... .......... .......... ..........
BOF       .......... .......... .......... .......... ..........
P89       .......... .......... .......... .......... ..........

Type I    LLPYDAVTVP TQPPFEELNP GQSNYPVANY FLLMPPPMMS LETLHRIVDH
Type III  .......... .......... .......... .......... ..........
Type II   .......... S......... .......... .......... ..........
GUY-DOS   .......... .......... .......... .......... ...F.....R
GUY-KOE   .......... .......... .......... .......... ...F.....R
VAND      .......... .......... .......... .......... ...F.....R
RUB       .......... .......... .......... .......... ...F.....R
GUY-MAT   .......... .......... .......... .......... ...F.....R
MAS       .......... .......... ..R....... .......... .........R
CASTELLS  .......... .......... ..R....... .......... .........R
COUGAR    .......... .......... .......... .......... .........R
GPHT      .......... .......... .......... .......... ..........
CAST      .......... .......... .......... .......... ..........
BOF       .......... .......... .......... .......... ..........
P89       .......... .......... .......... .......... ..........

Type I    EEFLTGDIGR VVRMVLTAEL IRIAANIQIR GLVHGRITSE NLFIMPDGRL
Type III  .......... .......... .......... .......... ..........
Type II   .......... .......... .......... .......... T.........
GUY-DOS   .......... .......... .......... .......... ..........
GUY-KOE   .......... .......... .......... .......... ..........
VAND      .......... .......... .......... .......... ..........
RUB       .......... .......... .......... .......... ..........
GUY-MAT   .......... .......... .......... .......... ..........
MAS       .......... .......... .......... .......... ..........
CASTELLS  .......... .......... .......... .......... ..........
COUGAR    .......... .......... .......... .......... ..........
GPHT      .......... .......... .......... .......... ..........
CAST      .......... .......... .......... .......... ..........
BOF       .......... .......... .......... .......... ..........
P89       .......... .......... .......... .......... ..........

Type I    MLGDVSALRK VGTRGPVSSV PVTYAPREFL SNTETAAFTH ALDAWQLGLV
Type III  .......... .......... .......... .......... ..........
Type II   .......... .......... .......... .......... ..........
GUY-DOS   .......... .......... .......... .......... ..........
```

```
GUY-KOE  .......... .......... .......... .......... ..........
VAND     .......... .......... .......... .......... ..........
RUB      .......... .......... .......... .......... ..........
GUY-MAT  .......... .......... .......... .......... ..........
MAS      .......... .......... .......... .......... ..........
CASTELLS .......... .......... .......... .......... ..........
COUGAR   .......... .......... .......... .......... ..........
GPHT     .......... .......... .......... .......... ..........
CAST     .......... .......... .......... .......... ..........
BOF      .......... .......... .......... .......... ..........
P89      .......... .......... .......... .......... ..........

Type I   IHRIWCLILP FGLVTPRMKR SGRRPSMRVP GLDVLSVEAC TPMPDAVEML
Type III .......... .......... .......... .......... ..........
Type II  .......... .......... .......... .......... ..........
GUY-DOS  .......... .......... .......... .......... ..........
GUY-KOE  .......... .......... .......... .......... ..........
VAND     .......... .......... .......... .......... ..........
RUB      .......... .......... .......... .......... ..........
GUY-MAT  .......... .......... .......... .......... ..........
MAS      .......... .......... .......... .......... ..........
CASTELLS .......... .......... .......... .......... ..........
COUGAR   .......... .......... .......... .......... ..........
GPHT     .......... .......... .......... .......... ..........
CAST     .......... .......... .......... .......... ..........
BOF      .......... .......... .......... .......... ..........
P89      .......... .......... .......... .......... ..........

Type I   VRHFLNFNTR KRLLPLTAMG TPEFRQLQLD ISTSLSSK*
Type III .......... .......... .......... .........
Type II  .......... .......... .......... .........
GUY-DOS  .......... .......... .......... .........
GUY-KOE  .......... .......... .......... .........
VAND     .......... .......... .......... .........
RUB      .......... .......... .......... .........
GUY-MAT  .......... .......... .......... .........
MAS      .......... .......... .......... .........
CASTELLS .......... .......... .......... .........
COUGAR   .......... .......... .......... .........
GPHT     .......... .......... .......... .........
CAST     .......... .......... .......... .........
BOF      .......... .......... .......... .........
P89      .......... .......... .......... .........
```
